# The LONI QC System: A Semi-Automated, Web-Based and Freely-Available Environment for the Comprehensive Quality Control of Neuroimaging Data

**DOI:** 10.3389/fninf.2019.00060

**Published:** 2019-08-28

**Authors:** Hosung Kim, Andrei Irimia, Samuel M. Hobel, Mher Pogosyan, Haoteng Tang, Petros Petrosyan, Rita Esquivel Castelo Blanco, Ben A. Duffy, Lu Zhao, Karen L. Crawford, Sook-Lei Liew, Kristi Clark, Meng Law, Pratik Mukherjee, Geoffrey T. Manley, John D. Van Horn, Arthur W. Toga

**Affiliations:** ^1^Laboratory of Neuro Imaging, USC Mark and Mary Stevens Neuroimaging and Informatics Institute, University of Southern California, Los Angeles, CA, United States; ^2^Department of Gerontology, University of Southern California, Los Angeles, CA, United States; ^3^Department of Radiology & Biomedical Imaging, University of California, San Francisco, San Francisco, CA, United States

**Keywords:** quality control, magnetic resonance imaging, diffusion tensor imaging, functional magnetic resonance imaging, LONI Pipeline

## Abstract

Quantifying, controlling, and monitoring image quality is an essential prerequisite for ensuring the validity and reproducibility of many types of neuroimaging data analyses. Implementation of quality control (QC) procedures is the key to ensuring that neuroimaging data are of high-quality and their validity in the subsequent analyses. We introduce the QC system of the Laboratory of Neuro Imaging (LONI): a web-based system featuring a workflow for the assessment of various modality and contrast brain imaging data. The design allows users to anonymously upload imaging data to the LONI-QC system. It then computes an exhaustive set of QC metrics which aids users to perform a standardized QC by generating a range of scalar and vector statistics. These procedures are performed in parallel using a large compute cluster. Finally, the system offers an automated QC procedure for structural MRI, which can flag each QC metric as being ‘good’ or ‘bad.’ Validation using various sets of data acquired from a single scanner and from multiple sites demonstrated the reproducibility of our QC metrics, and the sensitivity and specificity of the proposed Auto QC to ‘bad’ quality images in comparison to visual inspection. To the best of our knowledge, LONI-QC is the first online QC system that uniquely supports the variety of functionality where we compute numerous QC metrics and perform visual/automated image QC of multi-contrast and multi-modal brain imaging data. The LONI-QC system has been used to assess the quality of large neuroimaging datasets acquired as part of various multi-site studies such as the Transforming Research and Clinical Knowledge in Traumatic Brain Injury (TRACK-TBI) Study and the Alzheimer’s Disease Neuroimaging Initiative (ADNI). LONI-QC’s functionality is freely available to users worldwide and its adoption by imaging researchers is likely to contribute substantially to upholding high standards of brain image data quality and to implementing these standards across the neuroimaging community.

## Introduction

To ensure the highest standards of research quality, reliability, validity, and reproducibility in brain imaging studies, investigators who acquire and/or analyze neuroimaging data are required to test and monitor all facets of image acquisition. For this reason, image quality control (QC) is a prerequisite to most single and multisite projects. Acquisition protocols with relatively long scanning times, such as diffusion tensor imaging (DTI) and functional magnetic resonance imaging (fMRI), may be sensitive to substantial noise or artifacts during scanning – for instance, artifacts related to subject motion during relatively long duration acquisitions. Adherence to standardized protocol compliance may be inconsistent. Such neuroimaging challenges become more germane in imaging studies of children (Yoshida et al., 2013) and adolescents (Satterthwaite et al., 2012); the confounding influence of head motion on resting-state functional connectivity and DTI structural connectivity (Lauzon et al., 2013; Yoshida et al., 2013) have received substantial attention (Power et al., 2012; Satterthwaite et al., 2012; Van Dijk et al., 2012). Similar effects (Reuter et al., 2015) are evident in 3D acquisitions of structural MRI (sMRI).

In addition to head motion-induced artifacts, the common classes of artifacts found in MRI include ringing artifacts driven by aliasing, EPI distortions due to gradient effects, intensity inhomogeneity across regions due to MR strength attenuation and use of multiple channel coils, zero fill artifact, zipper artifact related to blood flow, impulse noise that likely drops the signal-to-noise ratio (SNR), magnetic susceptibility creating image geometric distortion (Bastin et al., 1998; Skare et al., 2000; Anderson, 2001), chemical shift due to the differences between resonance frequencies of fat and water (Reiser et al., 2008), and aliasing artifacts resulting from a field of view that is smaller than the object (Heim et al., 2004; Owens et al., 2012; Jones et al., 2013; Pizarro et al., 2016). Beyond the aforementioned artifacts, the quality of DTI measurement is also susceptible to eddy currents. These confounds likely contribute to inaccuracies in segmentation of anatomical MRI images (Pizarro et al., 2016; Keshavan et al., 2017), assessment of inter-regional correlation of blood-oxygen-level dependent (BOLD) time courses on resting state-fMRI (Power et al., 2012, 2014), and the tensor fitting of DTI data (Le Bihan et al., 2006). Poorly inspected data has the potential to obscure the presence of actual biological changes and/or produce spurious associations with study phenotypes. However, most neuroscientific and clinical studies do not describe whether or not image QC was performed in their research publications. Others rely solely upon a visual inspection method of image QC and follow in-house QC protocols, which may not be well documented. The use of visual inspection methods which often rely on subjective interpretation to identify ‘bad’ quality data are mainly due to the absence of an existing standardized procedure for QC. Furthermore, variations in QC approaches make data aggregation across datasets even more difficult.

Development of quantitative QC metrics is imperative for addressing the subjectivity in visual assessment and would serve to facilitate an automated QC system of brain image data so that methods of assessment can be reproduced across multisite datasets. A survey of the literature (Supplementary Data 1) presents studies performing systematic assessment of image quality of MRI data using quantitative QC for typical MRI modalities (sMRI: *n* = 9; fMRI: 5; DTI: 3). The types of QC (i.e., manual or fully automated annotation of ‘bad’ images), the number of QC metrics (*n* = 1–190) and the type of datasets (i.e., inclusion of patients or healthy subjects only, age range, sample size, use of publicly open data or their own data) used in these studies vary considerably. In particular, inclusion of pathologic brains or inclusion of pediatric or elderly groups in some studies may result in a different distribution of the QC metrics – suggesting different interpretations of their relative image quality since these are factors likely changing the degree of artifacts or degrading the image preprocessing for the computation of QC metrics. This may ultimately present confounds for the users during their interpretation of the QC results. Recent work shows more promising results and provides more advanced features that improve the accessibility and reliability of the QC system: The nine studies shown in Supplementary Data 1 focused on developing a QC system for structural MRI (sMRI). Similarly, these studies derived a number of QC metrics that characterize different aspects of imaging artifacts on sMRI and used supervised classifiers to determine a decision boundary by which the best agreement with visual inspection results was obtained. One of these frameworks is not publicly available (Pizarro et al., 2016). Roalf et al. (2016) recently developed a publicly open script which calculates several QC metrics to assess the image quality of DTI data. They performed a systematic evaluation on a large DTI dataset showing sensitivity and specificity of their proposed QC metrics to bad quality data. Oguz et al. (2014) have also developed the DTIPrep tool, open-source software featuring a graphical user interface (GUI), which can perform QC on DTI images. This tool has two separate modules including an automatic QC and artifact correction/removal as well as a module enabling visual assessment. One fMRI study using a QC metric of temporal variation in signal changes showed that this metric is sensitive to motion artifacts and also related to reductions in functional connectivity (Power et al., 2012). In their follow-up study, they expanded their findings by investigating methods to remove the censored motion artifact (Power et al., 2014). There have also been efforts made for the quality assurance of post-image processing such as in the studies evaluating brain structural segmentation on sMRI (Keshavan et al., 2017) and fiber tractography extracted from DTI data (Sommer et al., 2017). However, this type of QC processing may tend to be computationally costly, requiring numerous stages of image processing prior to the image quality evaluation.

Despite these recent efforts in various MRI modalities, several challenges exist which potentially limit neuroimaging researchers’ and clinicians’ access to or familiarity with currently available QC tools: First, There are no other comprehensive QC tools covering sMRI, fMRI, computed tomography (CT) and DTI simultaneously, even though there are other QC tools covering part of these image modalities^1^^,^^2^ (Marcus et al., 2013; Esteban et al., 2017). Second, most of the QC tools do not provide a user-friendly GUI which can increase the accessibility of novice-level users to these tools. Most tools also require preinstalled software libraries such as FSL, SPM or AFNI in order to enable their functionality on a local host computer. Furthermore, the facility for automated QC is not routinely included in many neuroimaging software packages, which potentially implies a dependence on human efforts in the QC process. Lastly, running a given QC tool on a personal computer or small size compute clusters may limit QC efforts in large-scale data collections.

Here, we describe the LONI QC system (version 1.0) which features a detailed scientific workflow for the objective review and assessment of various modality and contrast imaging data including sMRI, fMRI, DTI, and CT data. The current QC system has two options to perform its functionality: (1) a completely online system supported by various commonly-used web-browsers and which requires no preinstalled software; (2) a downloadable framework which runs on the user’s local computing environment but does necessitate prerequisite software. In the online system, the design allows users to anonymously upload imaging data to the LONI QC system, either through LONI Integrated Data Archive (IDA) or using a direct uploading interface. It computes a comprehensive set of standard QC metrics that have been described in the literature and performs a standardized QC via an automated pre-processing system specifically designed to generate a range of scalar and vector statistics along with derived images. LONI QC data processing workflows are implemented using the LONI Pipeline^3^ that facilitates designing, modifying, and maintaining the system, whilst the QC data processing is performed on the LONI processing grid in the Mark and Mary Stevens Neuroimaging and Informatics Institute at the University of Southern California (USC) – a cluster of thousands of central processing units (CPUs). LONI QC system also features a user-friendly web GUI that is designed for those whose level of expertise can range from novice to expert. Upon completion of the QC process, the system provides the users a detailed report containing a range of quantitative metrics which can be used to assess neuroimaging data quality. Finally, the LONI QC system enables image evaluation based on flagging each QC metric as ‘good,’ ‘questionable,’ or ‘bad’ based on a statistical distribution of prior results.

To provide an illustration of the LONI QC system, we evaluate various datasets including imaging data scanned with different imaging modalities (sMRI, fMRI, DTI), sequences (T1-weighted, T2-weighed, FLAIR) and different acquisition parameters (e.g., repetition time, echo time, voxel size). We also evaluate the QC metrics’ reproducibility (for a dataset collected from the same scanner and collected from multiple scanners with different acquisition parameters) as well as sensitivity and specificity to the identification of ‘bad’ quality images in comparison to visual inspection to assess the utility of the automated QC rating process.

## Materials and Methods

**FIGURE 1:**
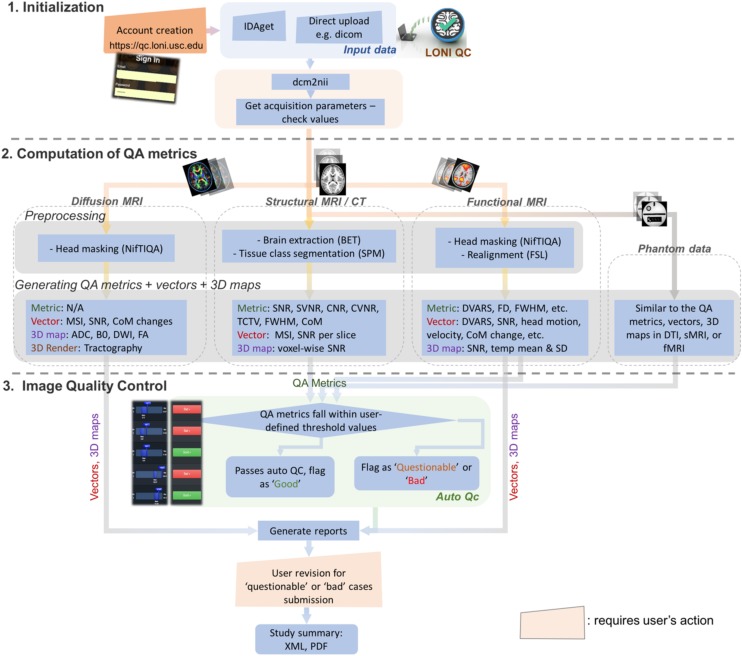
Overall workflow for the LONI QC system. The LONI QC system consists of the three main stages: **(1)** Initialization including the creation of an online account and uploading data; **(2)** Computation of QC metrics for different image modalities. The system computes and generates various QC metrics, vectors and 3D maps and renderings for user’s comprehensive evaluation of image quality; and **(3)** Image QC including automated QC and user’s visual evaluation. The automated QC feature provides a way for users to be informed about whether the assessed image data is of good quality or needs further careful inspection by a human expert. IDA, Integrated Data Archive; QC, quality assessment; QC, quality control; MSI, mean slice intensity; SNR, signal-to-noise ratio; CoM, Center of Mass; ADC, apparent diffusion coefficient; DWI, diffusion-weighted imaging; FA, fractional anisotropy; SVNR, signal variance-to-noise variance ratio; TCTV, tissue contrast-to-tissue variance ratio; DVARS, the root-mean-squared change in blood oxygenation level-dependent signal across time; FWHM, full width half maximum; FD, frame-wise displacement; SD, standard deviation.

**FIGURE 2:**
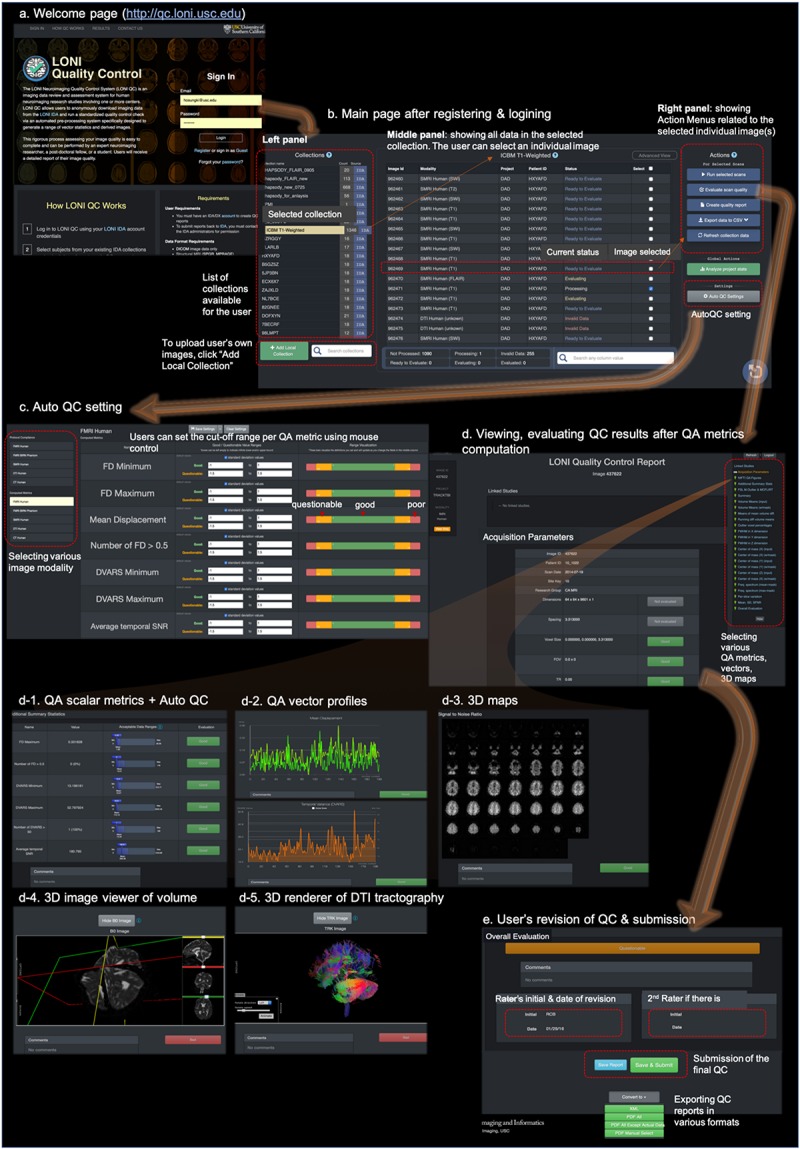
The web-based user-friendly GUI for LONI QC system. **(a)** Entering to http://qc.loni.usc.edu using any web-browser, users can register their accounts and log in to LONI QC system. **(b)** After sign-in, users enter into the main page. In the left panel the user can first select a data collection. The user can select image(s) in the selected collection in the middle panel. Finally, in the left panel, the user can select an Action related to the selected image(s): either run new QC, evaluate QC result, create QC report, export QC data to a CSV file or refresh collection data. **(c)** The user can set or change the cutoff values/ranges in ‘auto QC setting.’ The cutoff ranges are set per image modality by selecting it on the left-bottom panel. The ranges can be compared to the mean and SD of the previously processed datasets. **(d)** Once QC metrics computation were completed and the user clicked “Evaluate scan quality” in the left panel of the main page, the user can appreciate and evaluate the calculated QC metrics **(d-1)**, vectors **(d-2)**, and 3D maps and renderings **(d-3,4,5)** per image. If the auto QC was performed, they can find the ‘good,’ ‘questionable’ or ‘bad’ flags and can revise the results if they do not agree. After the evaluation and revision of QC, users can submit the final evaluation to the system and request to export the QC reports in various formats such as XML or PDF **(e)**.

The online LONI QC system consists of the following three stages (Figure 1): (1) Initialization, including online account creation and uploading data; (2) Computation of QC metrics for various modality images; and (3) Image QC reporting including automated QC rating and user’s visual inspection. The automated QC feature provides a way for users to be informed about whether the assessed image data is of good quality or needs further careful inspection by a human expert. In the following sections, the workflow and technical specifications of the system are described. More details that explain how the GUI of the current system interacts with the workflow and the proposed features are provided in Figure 2 and Supplementary Data 2.

The offline version of the LONI QC framework may be downloaded at http://qc.loni.usc.edu. This package includes the related LONI pipeline workflow file, the scripts required by the workflow, and a document instructing the installation and the list of the packages to be preinstalled, such as FSL, AFNI, FreeSurfer, and SPM.

### Initialization (Figure 1-1)

Once users create their account on the LONI QC system and login, they can submit image data from the existing data collection to the QC processing workflow. To enable the submission of image data by a user, the LONI QC system either interacts with the LONI-Image Data Archive (IDA)^4^ or uses a separate module that allows the user to directly upload their data to the QC system. The IDA is a user-friendly environment for archiving, searching, sharing, curating and disseminating neuroimaging and related clinical data (Crawford et al., 2016). It has been employed in a large number of neuroimaging research projects across the globe and accommodates MRI, MR angiography (MRA), magnetic resonance spectroscopy (MRS), DTI, CT, positron emission tomography (PET) and other imaging modalities. An engine for flexible data de-identification and encrypted file transmission are then used to ensure compliance with patient-privacy regulations. Uploading data through the IDA automatically archives the data in the IDA securely, which requires no specialized hardware, software or personnel. The IDA and the direct upload module automatically extract relevant metadata from all de-identified image files. The direct upload method implemented in the current system version (v1.0) permits DICOM and Nifti formats as well as uploading multiple files at a time (up to 2 gigabytes or up to 30 files).

### Computation of QC Metrics for Various Modality Images (Figure 1-2)

The users can initiate the system for computation of the QC metrics by selecting data included in the existing data collection. The LONI QC system uses a LONI Pipeline workflow (Rex et al., 2003) to pre-process image data prior to the calculation of QC metrics including correction for intensity inhomogeneity (Sled et al., 1998) and eddy current correction for the geometric distortion on DTI (Jezzard et al., 1998). Once the preprocessing is done, the system then inspects all XML header information and verifies that the data are suitable for analysis: e.g., whether the modality of the image is within the category of sMRI, fMRI, DTI or CT, and whether there is missing information about the imaging parameters. The results of this inspection are used as input to a module which either instructs the system to proceed with the calculation of metrics or transmits information to an error reporting module. We describe in the following sections how and what QC metrics are computed in each imaging modality.

#### Workflow and QC Metrics for sMRI and CT

The following QC metrics are computed: (1) mean slice intensity (MSI): AFNI software is used to calculate MSI, a vector representing the mean intensities for all the slices. A quick change in mean intensity at a slice compared to its previous one may indicate a quality issue; (2) SNR: The lower 10% of the intensity distribution is used to separate the image background from the head. The SNR is, then, computed as mean signal intensity of the head divided by the standard deviation (SD) of the intensity in the background. The range of possible values is between zero and infinity. Lower SNR indicates poorer image quality; (3) signal variance-to-noise variance ratio (SVNR): Signal intensity variance of the head is divided by the signal intensity variance of the background. Here, the range of possible values is between zero and infinity. Higher SVNR indicates bad image quality; (4) contrast-to-noise ratio (CNR): Image is skull stripped to label the brain using FSL-BET^5^. The FSL-BET is subject to the generation of a poorly fitting brain mask. However, we intend for the LONI QC system to use simple and minimum image pre-processing steps rather than employing learning-based approaches, *per se*, which often perform better or worse depending on the training-set. Furthermore, the LONI QC system has the functionality for users to visually check the quality of the BET-generated mask, allowing for the finalization of the QC more comprehensively. Segmentation of gray matter (GM), white matter (WM), and cerebrospinal fluid (CSF) is performed on the skull-stripped brain using SPM8^6^ package. The means of GM and WM signal intensities are subtracted from one another. Their absolute value is divided by the SD of the background signal intensity. Possible values range from zero to infinity. Lower CNR indicates poorer image quality; (5) Contrast of Variance-to-Noise Ratio (CVNR): Instead of the means of GM and WM intensities, their SDs are used; (6) brain tissue contrast-to-tissue intensity variation (TCTV): The means of GM and WM signal intensities are subtracted from one another. Their absolute value is divided by the pooled SD of the GM and WM as $\sqrt{\sigma_{G\,M}^{2}+\sigma_{W\,M}^{2}}$ where σ is SD of the signal intensities for a given tissue type. Range of values is zero to infinity. Smaller TCTV indicates poorer image quality. This metric was used in a recent study (Pizarro et al., 2016) and we observed this is sensitive to the motion artifact more than SNR or CNR; (7) full-width-at-half-maximum (FWHM): This metric that characterizes the smoothness of the image is determined using the variance of derivatives method of Worsley et al. (1992): The FWHM was computed within the brain area and calculated separately for each axis in the image volume. Also, the number of ‘resolvable elements’ is calculated by dividing the number of voxels in the brain by the geometric mean of the FWHM of each axis; (8) center of mass (CoM) of the volume in each dimension (X, Y, and Z). The CoM is computed by dividing the sum of each coordinate X, Y, or Z for the voxels inside the brain by the number of these voxels (Fesl et al., 2008).

#### Workflow and Metrics for DTI

The module first ensures whether data for all gradient directions are available or not by extracting *B*_*0*_ values and the diffusion gradient direction matrix from DICOM headers. If this is not the case, the workflow generates error messages to the user. The following QC vectors are computed: (1) the SNR; (2) CoM computed for each gradient direction volume; (3) histogram of image intensities and its related descriptive statistics for each volume are generated within the head mask; (4) the mean signal intensity (MSI) and SNR for volumes associated with each gradient direction; and (5) the displacement from the mean of the CoM in each of the X, Y, and Z directions is calculated for each gradient direction. Using TrackVis^7^, the following features as volume maps are computed and visualized for users to examine: *B*_0_, fractional anisotropy (FA), mean diffusivity (MD) and apparent diffusion coefficient (ADC) volumes. The 3D rendering of WM fibers is generated using streamline tractography methods (Mori et al., 1999; Lazar et al., 2003). A detailed report containing information about the number of voxels, mean intensity, standard deviation, and minimum and maximum intensities for each slice is also generated.

#### Workflow and Metrics for fMRI

For each point in an fMRI time series, capabilities are provided to calculate the following scalar metrics: (1) MSI per volume; (2) the average temporal SNR; (3) the frame-wise displacement (FD): the mean displacement of the head for each frame from the first frame volume using the algorithm of Power et al. (2012). The maximum FD and the number of the volume frames with FD > 0.5 are also computed; and (4) DVARS: The algorithm of Power et al. (2012) is also used to compute the root-mean-squared change in blood oxygenation level-dependent (BOLD) signal across time, which is known as the DVARS measure. We used FSL tools called fsl_motion_outliers to compute FD and DVARS. We further compute the maximum DVARS, the number of frames with DVARS > 50. Plots across time (i.e., across the volume frames) are also provided for the following quantities: (1) FD; (2) DVARS; (3) the volume mean of SNR; (4) estimated head translations and rotations in each dimension; (5) the volume mean of the signal intensity; (6) the volume mean of the running difference (‘velocity’); (7) percentage of outlier voxels [using the 3dToutcount function in the AFNI software package (Cox, 1996)]; (8) the FWHM in each dimension; (9) the CoM change in each dimension; (10) the mean and maximum of the fMRI signal’s frequency spectrum over the brain-masked volume; and (11) the image intensity variation per slice and the signal-to-fluctuation noise ratio (SFNR) computed as described by Friedman and Glover (2006). In the processes where the alignment was required, we used FSL-FLIRT and MCFLIRT tools with the cost function of the normalized correlation.

#### Workflow and Metrics for Phantoms

The LONI QC system accommodates data collected from MRI phantoms as a separate category and all the metrics described above for human data can be computed automatically for MRI phantoms as well. This process can be essential for helping the user to decide on acceptable values and ranges for metrics computed from human data. The QC protocol for phantoms is similar to that for each type of imaging (sMRI/CT, DTI or fMRI), with minor differences. The QC metrics reported for phantoms are the MSI, odd-even slice intensity differences, the SFNR, the CoM in each dimension, and to obtain plots of the raw fMRI signal and Fourier spectrum magnitude.

### Image QC (Figure 1-3)

#### User’s Qualitative and Quantitative QC of Image Data

Once the image data were processed and QC metrics have been computed, the system awaits the users’ evaluation. The graphical user interface (GUI) of LONI QC system then is provided for users’ visual inspection of the quality of images as well as their quantitative evaluation of QC metrics (Figure 2):

• Visual inspection: The GUI is fully integrated with the LONI Viewer based on a web-enabled neuroimage viewing engine. For sMRI volumes, the LONI viewer allows users to inspect neuroimaging slices in the axial, sagittal and coronal planes. For DTI volumes, a magnetic field gradient direction table is provided in addition to FA, MD and ADC images. DTI tractography files can be inspected using the LONI Viewer with an online 3D visualization module.
• Quantitative evaluation: Using the GUI of the system, the users can view and examine the resulting QC metrics as in value for the following metrics [sMRI: SNR, CNR, SVNR, CVNR, TCTV, COV, FHWM [x,y,z], CoM [x,y,z]; fMRI: average temporal SNR, maximum FD, number of frames with FD > 0.5, minimum DVARS, maximum DVARS, number of frames with DVARS > 50; DTI: N/A], as in graph plotting the vector of image arrays (sMRI), gradient volume series (DTI), and time series profiles (fMRI) for the following metrics: [sMRI: MSI; DTI: MSI per gradient volume, SNR changes, CoM change in each dimension; fMRI: FD, DVARS, volume mean of SNR, head translations, and rotations in each dimension, volume mean of signal intensity, volume mean of running difference (‘velocity’), percentage of outlier voxels, FWHM in each dimension, CoM change in each dimension, mean and maximum of the fMRI signal’s frequency spectrum over the brain-masked volume, image intensity variation per slice and signal-to-fluctuation noise ratio], and as in voxel-wise volume map (sMRI: SNR; DTI: SNR, B_0_, FA, MD, ADC; FMRI: SNR, temporal mean, temporal SD).

#### Automated QC and User’s Revision

It is almost impossible for users to perform image QC for all the data in instances of the analysis of large or multisite datasets. Even analyzing a smaller dataset, image QC for every single subject is time-consuming. To facilitate, the LONI QC system provides a user-friendly automated QC system that flags each scan with ‘good,’ ‘questionable’ or ‘bad’ and suggests the user to carry out an additional visual QC on those with ‘questionable’ or ‘bad’ flags. This feature is currently available for the sMRI and fMRI data where we have single-value QC metrics whereas the QC metrics for DTI are in a vector format. The system’s GUI provides the users a way to set a range for each QC metric, with which they can classify the resulting metric to the ‘good,’ or ‘bad’ category (note: ‘questionable’ is merged into either ‘good’ or ‘bad’ in autoQC, see Evaluation section). This is performed by comparing the location of each metric value with a user-defined cut-off range. Metrics whose values fall outside this interval are then labeled automatically as ‘bad.’ In the current study, the criteria for the classification of ‘good’ or ‘bad’ were determined compared to the visual QC as the gold standard. More specifically, the criteria were defined based on the cut-off values which we determined at the best performance in terms of (sensitivity + specificity)/2. More details and the best cut-off values used for the current version of autoQC are found in the Evaluation section. Finally, if more than a user-specified number (system default: 3) of computed metrics are flagged as ‘bad,’ the system flags the assessed case as ‘bad’ and suggests it to be more closely checked.

To finalize a QC report review and submission, the user provides an overall evaluation of the volume on the basis of the result of the auto QC as well as that of their own qualitative QC. The users can either accept the auto QC result or submit their revised annotation. Once reviewed, the report can be saved only, or saved and submitted to the QC database. If the report is only saved, additional changes can still be made until its final submission to the QC database. In each case, the users can convert the report into either PDF or CSV format for further download, distribution, or offline analysis. The entire QC process can be completed within less than a minute for each scan by an expert neuroimaging researcher who has been trained on how to use the system.

#### QC Study Summaries

One feature being provided by the QC system is the ability is to compile summaries of volume quality over a study or multiple studies with hundreds to thousands of participants, over particular acquisition types (sMRI, DTI, fMRI, etc.), over distinct project sites and over user-defined date ranges when the data were acquired.

### Evaluation of the QC Metrics and the Auto QC

To aid in a better understanding of the QC metrics used by the system and provide a guideline to set up the cutoff ranges for the auto QC, we performed the following evaluations with various datasets:

#### Distribution of QC Metrics in Data Collected Using a Single MRI Sequence

We computed the QC metrics on sMRI data (*n* = 642; age = 74 ± 8 years, 25–75% = 68–78 years) that have been collected in the Alzheimer’s Disease Neuroimaging Initiative (ADNI) using the same imaging parameter setting (T1-weighted Sagittal MP-RAGE; details found in Table 1). In the following analyses, we used the magnitude of each CoM and FWHM by computing $\sqrt{a_{x}^{2}+a_{y}^{2}+a_{z}^{z}}$, where *a* is either CoM or FWHM, instead of analyzing each of x, y, z directional metrics separately. To assess the distribution of the QC metrics, we plotted the histogram for each of them. The distributions characterized using the histogram were used as the reference in the following analysis of the data using the multi-sites multi-sequences. For fMRI, we analyzed 657 scans that were selected also from the ADNI project (age = 74 ± 7, 25–75% = 69–78), which were acquired using a single set of imaging parameters (ADNI Axial resting state fMRI protocol) shown in Table 2.

**TABLE 1:** Acquisition parameters for structural MRI of the ADNI dataset.

	**Alzheimer’s Disease Neuroimaging Initiative (ADNI)**
Sequence	Sagittal MP-RAGE/IR-SPGR
TR [ms]	7
TE [ms]	3
TI [ms]	400
Flip angle [degrees]	11
Matrix	256 × 256
Voxel size [mm^3^]	1 × 1 × 1
FOV [mm]	(260 – 270) × (252 – 262)
Number of axial slices	176 – 196
Number of scans	642

*TR, repetition time; TE, echo time; TI, inversion time; FOV, field of view; MP-RAGE, magnetization-prepared rapid acquisition gradient echo; SPGR, spoiled gradient recalled echo.*

**TABLE 2:** Acquisition parameters for the two different resting state-fMRI dataset: ADNI represents data acquired using a single set of imaging parameters whereas Track-TBI represents dataset acquired using various parameters from multi-sites for the cross-validation.

	**ADNI**	**Track-TBI**
Sequence	ADNI2 Axial resting- state fMRI	Axial Resting State fMRI
TR [ms]	3000	3000 – 3671
TE [ms]	30	30
TI [ms]	N/A	N/A
Flip angle [degrees]	80	80
Matrix	64 × 64	(60 – 480) × (64 – 512)
Voxel size [mm^3^]	3.3 × 3.3 × 3.3	(2.8 – 3.4) × (2.75 – 3.4) × (1 – 3.4)
FOV [mm]	212 × 206	(64 - 512) × (62 – 497)
Number of axial slices	48	39 – 52
Number of frames	140	140, 200
Number of scans	657	1555

*TR, repetition time; TE, echo time; TI, inversion time; FOV, field of view.*

#### Reproducibility of QC Metrics on Data Collected From Multi-Sites, From Different Scanners and Using Multi-MRI Sequences

For sMRI, we used multisite datasets including data from Parkinson’s Progression Markers Initiative (PPMI) (Kang et al., 2016) and Transforming Research and Clinical Knowledge in Traumatic Brain Injury (TRACK-TBI) projects while using the ADNI data as the reference of the single sequence imaging data. For TRACK-TBI data, we included only those with non-visible injury on images in the analysis. As a result, we analyzed 1196 T1-weighted imaging data from PPMI (age = 62 ± 10, 25–75% = 56–69) and 1569 from TRACK-TBI projects (age = 37 ± 17, 25–75% = 24–52). For fMRI, we analyzed 1555 from TRACK-TBI (age = 37 ± 16, 25–75% = 24–52). The information of MRI acquisition parameters used in these sMRI and fMRI datasets are presented in Tables 2, 3.

**TABLE 3:** Acquisition parameters for the multi-site datasets used for the cross-validation: structural MRI.

	**Track-TBI**		**PPMI**	
**Sequence**	**Sagittal 3D T1 MPRAGE / 3D T1 IR-SPGR**		**Sagittal 3D T1 MPRAGE or 3D T1 IR-SPGR**	
TR [ms]	4 – 35	1160 – 2530	5 – 11	1650 – 2400
TE [ms]	1 – 8	2 – 20	2 – 6	2 – 20
TI [ms]	400 – 750	500 – 1300	400 – 500	844 – 1100
Flip angle [degrees]	8 – 30	7 – 160	8 – 30	8 – 160
Matrix	(224 – 512) × (256 – 512)	(204 – 512) × (245 – 512)	(256 – 512) × (160 – 512)	(192 – 560) × (192 – 560)
Voxel size [mm^3^]	(0.4 – 1.4) × (0.4 – 1.4) × (0.5 – 3)	(0.4 – 1) × (0.4 – 1) × (0.5 – 3)	(0.4 – 1.2) × (0.4 – 1) × (0.7 – 2)	(0.4 – 1.3) × (0.4 – 1.3) × (0.5 – 3)
FOV [mm]	(220 – 350) × (2134 – 340)	(220 – 260) × (214 – 252)	(160 – 266) × (155 – 258)	(220 – 270) × (214 – 262)
Number of axial slices	60 – 336	64 – 208	72 – 256	72 – 240
Number of scans	800	769	286	910
Scanner name (*n*)	Simens Triotrim (655) Simems Skyra (145)	Philips Achiava (531) GE Signa-HDXT (238)	N/A	N/A

*TR, repetition time; TE, echo time; TI, inversion time; FOV, field of view; MP-RAGE, magnetization-prepared rapid acquisition gradient echo; SPGR, spoiled gradient recalled echo; N/A, not available.*

To assess the distribution of the QC metrics, we plotted the histogram for each of them. For each modality of sMRI or fMRI, we created the histogram separately for each of the two datasets and compared the distribution of each metric between the two datasets. To this end, we first computed the *z*-score per QC metric using the pooled datasets of the two datasets. Then, the histogram in each dataset was normalized using the same number of the bins and by dividing the height of each bin by the area of the histogram, resulting in an empirical probability density map. Finally, to evaluate whether the manufacturer of the scanner affect the distribution of the QC metrics, we compare the histogram of the QC metrics measured in the subjects scanned on the Siemens scanner which comprised the major portion (*n* = 655; 42%; more information in Table 3) of the TRACK-TBI dataset with those measured in the whole TRACK-TBI dataset.

More subject motion is presumed to be involved in pediatric samples. Furthermore, more CSF volume, less cortical GM volume and smaller GM/WM tissue intensity contrast are expected in elderly (Steen et al., 1995; Salat et al., 2009) and dementia populations (Westlye et al., 2009; Salat et al., 2011). These factors possibly influence the measurement of the QC metrics. Thus, we correlated the age at scanning and each QC metrics. Visual inspection of the shape for each dataset’s probability density map and computing the Dice overlap index between them assessed their similarity.

Finally, a user may expect one or a combination of QC metrics to characterize a different aspect of the image artifacts. To evaluate the independency of a given QC metric to others for each modal image data, we constructed a matrix, each component of which computed a Pearson’s correlation efficient between the given metric and one of the rest of the metrics.

#### Reproducibility of QC Metrics for the Cases Scanned on the Same Scanner With the Same MRI Protocol

Four healthy volunteers, as well as a BIRN MRI phantom (Friedman and Glover, 2006), were scanned at 1-week intervals for a month (four scans) using the ADNI3 (Weiner and Veitch, 2015) neuroimaging protocol. This consisted of (A) structural MRI scans, including (i) a magnetization-prepared rapid acquisition gradient echo (MP-RAGE) *T*_1_-weighted scan, (ii) a spoiled gradient-echo (SPGR) *T_2_^∗^*-weighted scan and (iii) a fluid-attenuated inversion recovery (FLAIR) scan, (B) a 126-direction DTI scan, and (C) an fMRI scan. The acquisition parameters for each of these are listed in Table 4. These volumes were acquired using the 3 T Siemens Prisma MRI scanner at the Mark and Mary Stevens Neuroimaging and Informatics Institute. All volunteers scanned in the single MRI machine provided written informed consent and the study was undertaken with the approval of the Institutional Review Board at the Keck School of Medicine of USC and according to the Declaration of Helsinki. The ages of the volunteers were 24, 25, 25, and 35; all were right-handed and healthy, with no history of a neurologic or psychiatric disease. We expected a very small variability in the QC metrics across these images which were acquired in the same scanner relative to data collected from different scanners with different image sequences. We thus performed an *F*-test of Variance on a ratio as SD_multi_scanner^2^/SD_single_scanner^2^, by comparing the variance of each metric for these four individual images with the variance for the multi-site datasets mentioned above. This sample was created by consisting of only subjects in the same range of age as the four volunteers. The smaller the ratio SD_within_scanner/SD_multi_scanner was, the more reproducible the QC metrics were within a scanner.

**TABLE 4:** Acquisition parameters for the four healthy volunteers and 1 phantom scanned using the 3T Siemens Prisma MRI scanner at the Mark and Mary Stevens Neuroimaging and Informatics Institute.

	**sMRI**			**DTI**	**fMRI**
Weighting	T_1_	T_2_^∗^	FLAIR	T_2_^∗^	N/A
Sequence	MP-RAGE	SPGR	SE	EPI	FSE EPI
TR [ms]	2300.00	650.00	4800.00	3400.00	607.00
TE [ms]	2.95	20.00	441.00	71.00	32.00
TI [ms]	900.00	N/A	1650.00	N/A	N/A
Flip angle [degrees]	9	20	120	90	50
ETL	1	1	243	87	88
Acquisition type	3D	2D	3D	2D	2D
Matrix size	256 × 240	256 × 192	256 × 256	116 × 116	88 × 88
In-plain voxel size [mm]	1.05 × 1.05	0.86 × 0.86	1.00 × 1.00	1.00 × 1.00	2.50 × 2.50
Slice thickness [mm]	1.2	4.0	1.2	2.0	2.5
Phase FOV [%]	93.75	100.00	100	100	100
Bandwidth [Hz/pixel]	240	200	850	2270	2365

*The imaging parameters used in this acquisition were chosen in accordance with ADNI3 protocol. sMRI, structural magnetic resonance imaging; DTI, diffusion tensor imaging; fMRI, functional MRI; MP-RAGE, magnetization-prepared rapid acquisition gradient echo; SPGR, spoiled gradient recalled echo; SE, spin echo; EPI, echo planar imaging; FSE, fast SE; TR, repetition time; TE, echo time; TI, inversion time; ETL, echo train length; FOV, field of view; 2D, two-dimensional; 3D, three-dimensional.*

#### Performance of Auto QC

We assessed the performance of the auto QC in comparison to the result of the visual inspection. To find the best cutoff values as well as compare these values with the human visual QC results, we used the sMRI data of the TRACK-TBI dataset and tested various cutoff values to identify the QC labels (‘good’ vs. ‘bad’) that best agreed with the labels created by systematically performed expert’s visual inspection. Here, we tested only sMRI data as visual inspection of sMRI was performed solely using the evaluation of the original images without checking QC metrics. Visual inspection of fMRI normally entails the examination of the QC metrics as well, which could bias the inspection result. Furthermore, no scalar QC metrics were calculated for DTI data and thus the auto QC of DTI was not included in the current system. For visual inspection, we used the following categories of the artifact to identify ‘questionable (or moderate)’ and ‘bad’ quality images: ringing artifacts due to motion or aliasing, zipper artifact related to blood flow, impulse noise that likely drops the SNR, magnetic susceptibility creating image geometric distortion, wrap around artifacts happening when the size of the imaged object is larger than the field of view and small head coverage. The details of the visual inspection are provided in Supplementary Data 3. Using this protocol and being independent of the auto QC results, one rater (HT) labeled 1,569 individual t1-weighted sMRI data in the TRACK-TBI set and another rater (RECB) did this for a randomly subsampled 100 cases to test their reproducibility. The ‘questionable’ quality data in the visual assessment were either merged to ‘good’ or ‘bad.’

To assess the binary classification accuracy of each QC metric with respect to various cutoff values, we first changed the cutoff values per QC metric from *z*-score = −5 to *z*-score = 5 with a very small step size (*z*-score = 0.05). To compute sensitivity and specificity compared to expert labeling, we calculated the receiver operating characteristics (ROCs) and the related area under the curve (AUC). The logistic ROC analysis used a threefold cross-validation approach to estimate AUC and optimal cutoff score that resulted in the greatest accuracy as ‘(sensitivity + specificity)/2′. Larger AUC values indicated the more accurate classification of participants.

In the Auto QC, more than a user-specified number of computed metrics were flagged as ‘bad’ and the system flagged the assessed case as ‘bad.’ Therefore, we assessed how many ‘bad’ flagged QC metrics should be used to best agree with the labels in the visual inspection. Using the optimal cutoff values that were determined previously, we flagged all the 7 QC metrics either into ‘good’ or ‘bad’ and counted the number of the ‘bad’ labeled metrics per image. At each threshold from 1 to 7, we computed the specificity, sensitivity, and accuracy compared to the visual inspection results.

All *p*-values were corrected using Bonferroni adjustment.

## Results

### Processing Time

The processing times for the preprocessing (e.g., brain masking) and calculation of QC metrics (mean ± SD) were approximately 7 min for sMRI, 6 min for CT, 8 min for fMRI and 4 min for DTI on a single Intel i7 CPU. Including the queuing process and possible network traffics, the average computational times were 22.8 ± 6.6 min for sMRI, 18.5 ± 5.9 min for CT, 16.0 ± 4.2 min for fMRI and 7.6 ± 2.2 min for DTI.

### Distribution of QC Metrics in Data Collected Using a Single MRI Sequence

**FIGURE 3:**
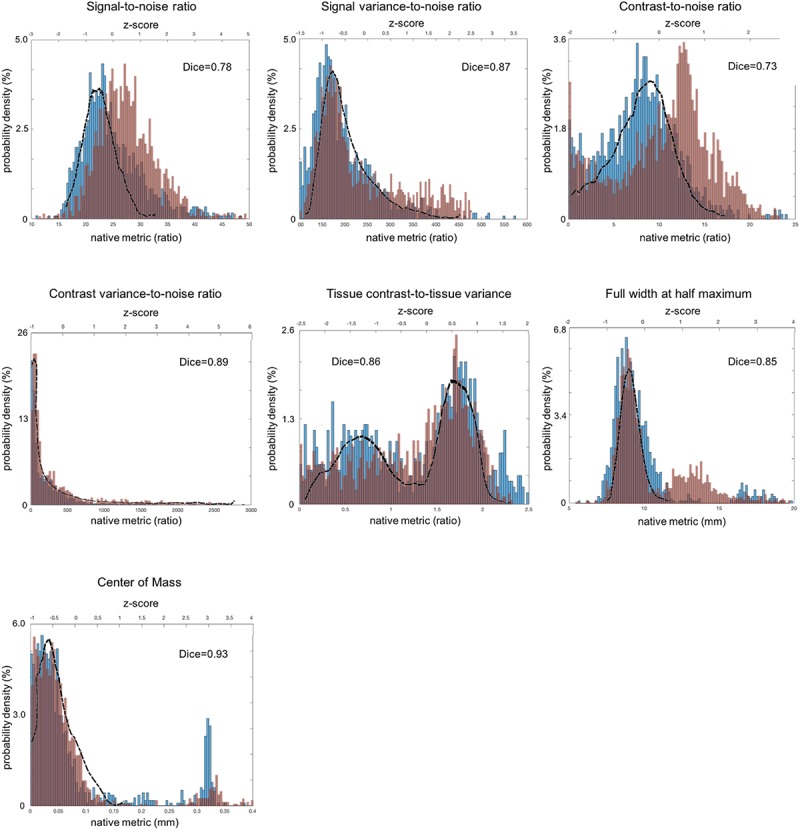
Distribution of sMRI QC metrics for two different datasets that were acquired with multiple imaging parameter settings and collected from multi-sites. The PPMI dataset is colored in blue and the TRACK-TBI in red while the ADNI dataset that was acquired using a single imaging parameter setting is used a reference and shown with the black outline. All the images included in this analysis were based on T1-weighted acquisition (The image sequence parameters are described in Table 3). The Dice similarity index was computed for each QC metric to evaluate the overlap between the distributions from the two multi-sites datasets. This was used as a measure of reproducibility of the metrics. Dice index: 0.6–0.8 – good; 0.8–1.00 – excellent (Altman, 1999).

For sMRI, the histogram of each QC metric is shown in Figure 3. Their mean and SD were: SNR = 21.4 ± 3.0; SVNR = 233 ± 56; CNR = 7.45 ± 3.33; CVNR = 788 ± 1680; TCTV = 1.00 ± 0.58; FWHM = 5.30 ± 0.3; CoM = 17.4 ± 3.8. A visual evaluation found that the distribution of SNR, SVNR, CNR, and FWHM was left-right symmetric and similar to the shape of a Gaussian function whereas that of CVNR and CoM was skewed and close to the shape of an F-distribution function. The distribution of TCTV displayed with two modes and was like the function of a bimodal Gaussian mixture function.

**FIGURE 4:**
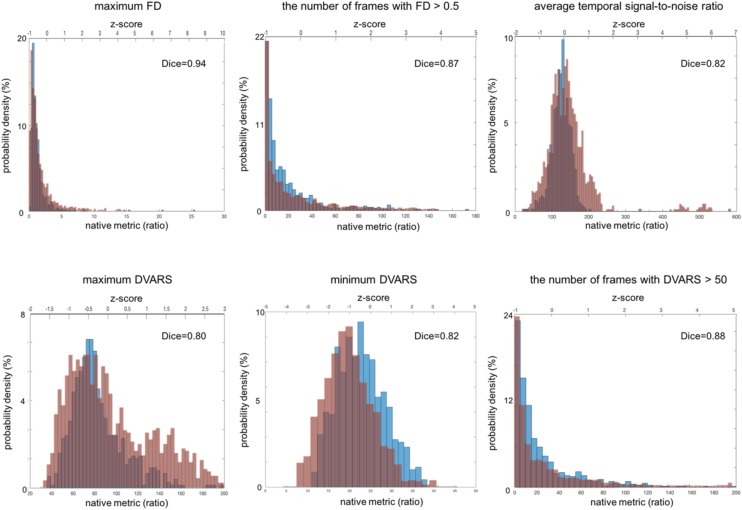
Distribution of fMRI QC metrics for two different multi-site data. The ADNI set that was acquired using a single imaging parameter setting is colored in blue while the TRACK-TBI set that was acquired using multiple imaging parameter settings is in red. All the images included in this analysis were based on Axial Resting State fMRI sequence (details in **Table 3**). The Dice similarity index was computed for each QC metric to evaluate the overlap between the distributions from the two datasets. This was used as a measure of reproducibility of the metrics.

For fMRI, the histograms are shown in Figure 4. The mean and SD of each QC metic were: maximum FD (maxFD) = 1.60 ± 8.80; the number of frames with FD > 0.5 (FD > 0.5) = 17.4 ± 24.3; average temporal signal-to-noise ratio (avgTSNR) = 126 ± 31; maximum DVARS (maxDVARS = 83.4 ± 37.4; minimum DVARS (minDVARS) = 23.0 ± 6.1; the number of frames with DVARS > 50 (DVARS > 50) = 21.9 ± 28.4. The distribution of avgTSNR, and minDVARS tended to be left-right symmetric and similar to the shape of Gaussian function whereas that of maxDVARS, maxFD, FD > 0.5 and DVARS > 50 was skewed. The estimated FWHM along each of the x, y, and z axes for fMRI was included in the system. However, because the resultant measurement is not a scalar but a time series vector, we did not include this in the result because of the complexity of the time-series vector metric.

### Reproducibility of QC Metrics for Data Acquired on a Scanner Using a Single Imaging Sequence

#### sMRI

All the individual QC metrics computed for the four volunteers’ longitudinal scans are provided in Tables 5–7. The means of all the QC metrics for the T1w MRI data were similar to those computed using the ADNI dataset and the multi-site PPMI and TRACK-TBI datasets whereas the variations for these single-scanner-acquired data were significantly smaller than those acquired from the multiple sites (*F*-test; *F* > 19; *p* < 0.00001). The distribution of each metric did not differ among the four individuals (ANOVA; *F* < 2.0; *p* > 0.3). The computation of the QC metrics in T2^∗^ and FLAIR imaging data showed different characteristics of their means and SDs compared to T1-weighted data (paired *t*-tests; *t* > 3.7; *p* < 0.05), advising the choice of different cutoff values in the auto QC setting depending on the used acquisition sequence. As expected, the mean SNR for the phantom was approximately 3–5 times higher for all three sequences. Similar differences between human subjects and the phantom were observed for the SVNR and FWHM.

**TABLE 5:** QC metrics for T_1_-weighted sMRI scans.

	**SNR**	**SVNR**	**CNR**	**CVNR**	**TCTV**	**FWHM**	**CoM**
S1	29.3 ± 0.5	251 ± 60	12.5 ± 0.1	83 ± 61	1.99 ± 0.34	10.7 ± 0.1	0.16 ± 0.00
S2	31.8 ± 3.4	316 ± 52	10.3 ± 6.0	148 ± 111	1.44 ± 0.55	11.0 ± 0.2	0.18 ± 0.01
S3	30.6 ± 0.8	260 ± 17	15.9 ± 6.8	374 ± 52	1.44 ± 1.06	10.4 ± 0.1	0.17 ± 0.01
S4	30.9 ± 1.8	259 ± 28	14.4 ± 0.6	140 ± 112	1.93 ± 0.91	11.2 ± 0.2	0.15 ± 0.01
All	30.7 ± 1.7	272 ± 26	13.3 ± 3.4	184 ± 83	1.70 ± 0.78	10.8 ± 0.1	0.17 ± 0.01
P	125.9 ± 2.5	569 ± 30	–	–	–	27.8 ± 0.4	0.13 ± 0.01

*Shown are the mean μ and standard deviation σ for each subject (S1, S2, S3, and S4) as well as for the phantom (P). CNR, CVNR and TCTV for the phantom were not computed as GM/WM tissue contrast is not defined.*

**TABLE 6:** QC metrics for T_2_^∗^ sMRI scans.

	**SNR**	**SVNR**	**CNR**	**CVNR**	**TCTV**	**FWHM**	**CoM**
S1	26.1 ± 0.3	214 ± 4	2.2 ± 0.9	343 ± 32	0.21 ± 0.09	21.3 ± 0.3	0.19 ± 0.01
S2	30.5 ± 0.2	285 ± 5	1.1 ± 0.9	335 ± 56	0.09 ± 0.08	21.2 ± 0.5	0.20 ± 0.00
S3	28.7 ± 1.5	252 ± 18	1.3 ± 0.6	235 ± 34	0.11 ± 0.05	22.7 ± 0.2	0.19 ± 0.01
S4	25.9 ± 0.7	243 ± 10	1.0 ± 0.5	475 ± 80	0.08 ± 0.04	20.9 ± 0.3	0.18 ± 0.01
All	27.8 ± 0.7	248 ± 9	1.4 ± 0.7	347 ± 51	0.12 ± 0.07	21.5 ± 0.3	0.19 ± 0.01
P	107.5 ± 2.1	582 ± 17	–	–	–	35.9 ± 0.5	0.19 ± 0.01

*Shown are the mean μ and standard deviation σ for each subject (S1, S2, S3, and S4) as well as for the phantom (P). CNR, CVNR and TCTV for the phantom were not computed as GM/WM tissue contrast is not defined.*

**TABLE 7:** QC metrics for FLAIR sMRI scans.

	**SNR**	**SVNR**	**CNR**	**CVNR**	**TCTV**	**FWHM**	**CoM**
S1	21.4 ± 0.5	154 ± 12	8.6 ± 2.2	43.5 ± 17.7	1.31 ± 0.40	8.81 ± 0.27	0.14 ± 0.01
S2	25.7 ± 0.9	212 ± 14	13.2 ± 0.3	34.1 ± 2.1	1.81 ± 0.04	9.59 ± 0.29	0.16 ± 0.01
S3	25.0 ± 0.8	199 ± 16	11.0 ± 0.4	19.4 ± 4.1	1.66 ± 0.08	9.81 ± 0.20	0.15 ± 0.00
S4	23.2 ± 1.2	196 ± 19	11.7 ± 0.9	34.3 ± 3.4	1.61 ± 0.20	9.73 ± 0.22	0.14 ± 0.01
All	23.8 ± 0.8	190 ± 15	11.1 ± 0.9	32.8 ± 6.8	1.60 ± 0.18	9.49 ± 0.24	0.15 ± 0.01
P	67.0 ± 3.9	186 ± 36	–	–	–	18.3 ± 0.6	0.13 ± 0.01

*Shown are the mean μ and standard deviation σ for each subject (S1, S2, S3, and S4) as well as for the phantom (P). CNR, CVNR and TCTV for the phantom were not computed as GM/WM tissue contrast is not defined.*

#### fMRI

The computed QC metrics are shown in Table 8. As in sMRI, their means were similar to those computed using TRACK-TBI and ADNI datasets except minimum DVARS and maximum DVARS (*t* > 4.2; *p* < 0.005). The variations for all the metrics were significantly smaller (*F*-test; *F* > 15; *p* < 0.00001). Results illustrated that, as expected, the temporal SNR was four times higher in the phantom whereas the FD and DVARS values were many times larger in human subjects (*t* > 21; *p* < 0.00001). This was because both FD and DVARS reflect greater subject motion, such that larger values are associated with more motion during the scan.

**TABLE 8:** QC metrics for fMRI.

	**max (FD)**	**No. FD > 0.5**	**max (DVARS)**	**min (DVARS)**	**No. DVARS > 50**	**Temporal SNR**
S1	0.868 ± 0.591	11.4 ± 14.0	104.2 ± 18.9	30.7 ± 0.8	20.2 ± 10.1	101.8 ± 7.6
S2	0.57 ± 0.318	6.2 ± 7.7	106.9 ± 64.3	29.4 ± 4.9	16.0 ± 11.3	128.9 ± 13.8
S3	0.505 ± 0.118	7.7 ± 7.2	76.7 ± 11.2	36.1 ± 1.7	11.3 ± 15.6	112.4 ± 5.9
S4	2.38 ± 0.648	14.0 ± 12.3	160.8 ± 31.2	33.9 ± 1.6	15.6 ± 17.6	88.5 ± 11.7
All	1.08 ± 0.419	11.1 ± 13.5	112.2 ± 31.4	32.6 ± 2.3	20.1 ± 18.5	108.0 ± 9.8
P	0.047 ± 0.011	0.05 ± 0.01	13.7 ± 0.9	13.1 ± 0.9	0.05 ± 0.01	438.5 ± 40.1

*Shown are the mean μ and standard deviation σ for each subject (S1, S2, S3, and S4) as well as for the phantom (P). SNR averages are computed across time. FD values are multiplied by 100 to facilitate comparison.*

### Reproducibility of QC Metrics for Multi-Site and Multi-Scanner Data

#### sMRI

All the distributions of the sMRI QC metrics are shown in Figure 3. The overall shapes of the histogram for all the metrics were similar between the PPMI and ADNI single sequence datasets. The distributions in all the QA metrics of PPMI data were well overlapped with those in the ADNI data, whereas the distributions of SNR and CNR in TRACK-TBI data displayed a shift of the whole shape from the PPMI and ADNI data, driven by their higher mean (SNR: +3.1; CNR = +4.2). Indeed, TRACK-TBI data displayed significantly higher mean SNR (26.6 ± 6.0 vs. 27.8 ± 5.8; *t* = 4.6; *p* < 0.001), and higher mean CNR (27 ± 0.1 vs. 27 ± 0.1; *t* = 4.8; *p* < 0.001) than PPMI data. No other QC metrics differed in their means (*p* > 0.2).

The overlap between PPMI and TRACK-TBI datasets was generally very high across metrics (Dice index: μ ± σ = 0.88 ± 0.03, range: 0.85–0.93) except SNR and CNR (0.76 ± 0.04) that displayed relatively smaller overlap. The largest overlap was observed in CoM (Dice index = 0.93), followed by CVNR (0.89), FWHM (0.87), TCTV (0.86), SVNR (0.85), SNR (0.78) and CNR (0.73), respectively. Despite the high overlap of the main distribution between TRACK-TBI and PPMI datasets, the FWHM displayed significant smaller peaks unequally located in the right-hand tail for both data sets. We found this was driven by a number of cases with artifacts.

The overlap between Siemens data of the TRACK-TBI and the whole TRACK-TBI data was also high across all the metrics (Dice index: μ ± σ = 0.90 ± 0.05, range: 0.73–0.93) except CoM (0.73) that displayed relatively smaller overlap. The largest overlap was observed in CVNR (Dice index = 0.95), followed by SVNR (0.89), CNR (0.87), TCTV (0.86), SNR (0.85), FWHM (0.82) and CoM (0.73), respectively (Supplementary Data 4).

Analysis of the age at scanning showed no correlation of any QC metric with aging in any dataset (Pearson’s correlation coefficient: *r* < 0.2; *p* > 0.1). Subgrouping the TRACK-TBI data into the pediatric (<20 years, *n* = 220) and adult (>20 years, *n* = 1349) groups did not display a difference in any QC metric (*t* < 1.0; *p* > 0.4) between these two groups. However, subgrouping the TRACK-TBI data into the elderly (>60 years, *n* = 260) and non-elderly (<60 years, *n* = 1309) showed a significant drop-down in SNR and CNR in the elderly group relative to the non-elderly (*t* > 4.7; *p* < 0.001). The mean of SNR and CNR in the elderly group of TRACK-TBI did not differ from those computed in PPMI or ADNI dataset (*t* < 1.3; *p* > 0.3). A subsequent investigation found that the lower SNR in the elderly than in the non-elderly group was driven by a significantly lower mean signal intensity within the head (the numerator of SNR; *t* = 6.1; *p* < 0.0001) while a variance of intensity in the background (the denominator of SNR) did not differ between the two age groups (*F* = 1.4; *p* > 0.1). The lower CNR in the elderly was due to a lower mean tissue contrast (the numerator of CNR; *t* = 10; *p* < 0.00001) while the variance of brain intensity (the denominator) was not different between the elderly and non-elderly group.

#### fMRI

All the distributions of the fMRI QC metrics are shown in Figure 4. The overall shapes of the histogram for all the metrics were also very similar between the TRACK-TBI dataset with multiple settings of imaging parameters and ADNI dataset with a single setting of imaging parameters. The overlaps between these two datasets were very high (Dice index: μ ± σ = 0.86 ± 0.05, range: 0.80–0.94). The largest overlap was observed in maxFD (Dice index = 0.94), followed by DVARS > 50 (0.88), FD > 0.5 (0.87), avgTSNR (0.82), minDVARS (0.82), and maxDVARS (0.80), respectively. The mean and the variance of each metric did not significantly differ between ADNI data than TRACK-TBI (*p* > 0.1). There was no correlation between any QC metric and the age in either of the two groups (*r* < 0.2; *p* > 0.1).

### Association of a Given QC Metric With Other Metrics

#### sMRI (Figure 5A)

Analysis of the correlations between a given QC metric and others in the pooled dataset of TRACK-TBI and PPMI sets showed that most of metrics were not significantly associated (*r* < 0.5; *p* > 0.1) whereas the following pairs were highly correlated: SNR-SVNR, CNR-TCTV, and CoM-FWHM (*r* > 0.5; *p* < 0.05). The reason for their significant correlation was likely due to that SNR and SVNR used the same denominator; CNR and TCTV used the same numerator and; CoM and FWHM characterized similarly about the head shape: i.e., the position and the blurriness.

**FIGURE 5:**
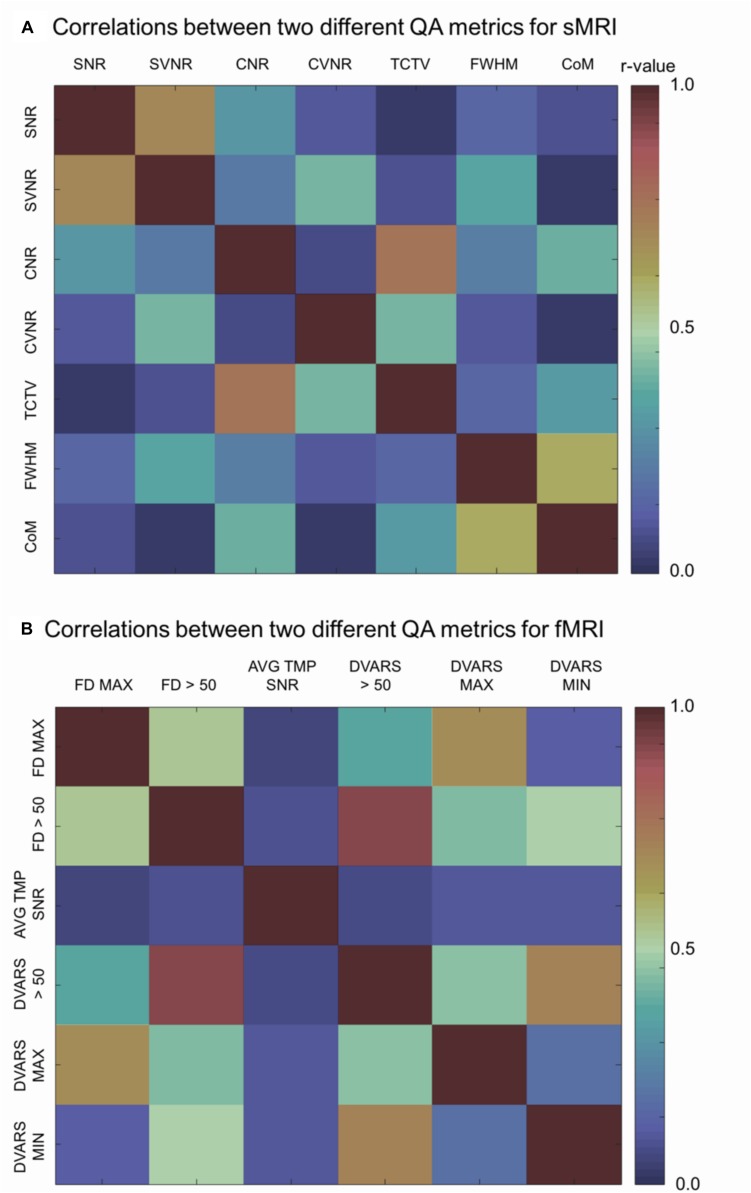
Correlation matrices. Each cell indicates the Pearson’s correlation coefficient computed between two indicated QC metrics. SNR, signal-to-noise ratio; SVNR, signal variance-to-noise ratio; CNR, contrast-to-noise ratio; CVNR, Contrast variance-to-noise ratio; TCTV, tissue contrast-to-tissue (intensity) variance; FWHM, full width-at-half maximum; CoM, center of mass; FD MAX, maximum Frame-wise displacement (FD); FD > 0.5, the number of frames with FD is larger than 0.5 mm; AVG TMP SNR, average temporal SNR; DVARS, the root-mean-squared change in blood oxygenation level-dependent signal across time; DVARS > 50, the number of frames with DVARS > 50; DVARS MAX, maximum DVARS; DVARS MIN, minimum DVARS.

#### fMRI (Figure 5B)

Analysis of the correlations between a given QC metric and others in the pooled dataset of TRACK-TBI and ADNI sets showed that the following pairs were highly correlated: maxFD-FD > 0.5, maxFD-maxDVARS, FD > 0.5-maxDVARS, FD > 0.5-minDVARS, DVARS > 50-FD > 50, DVARS > 50-maxDVARS, and DVARS > 50-minDVARS, (*r* > 0.3; *p* < 0.05). The avgTSNR did not correlate with any other metrics (*r* < 0.12; *p* > 0.2).

#### Evaluation of the Auto QC System

**FIGURE 6:**
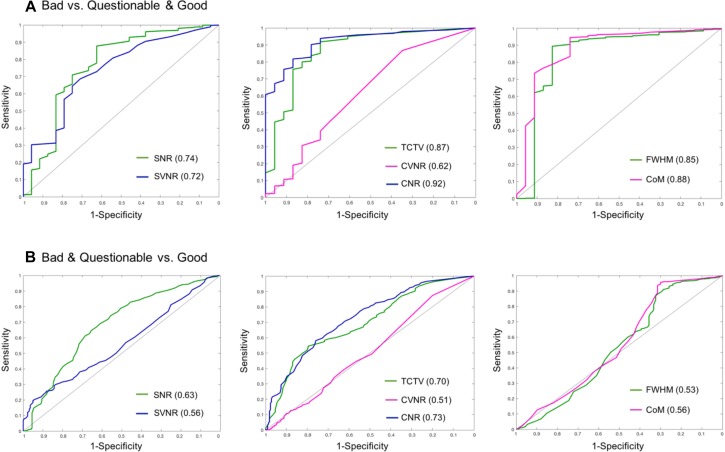
Receiver–operator characteristic (ROC) curves based on using sMRI QC metrics for classification. **(A)** ROCs for differentiating bad data from acceptable data (questionable and good). CNR best differentiated bad data from the acceptable data as it yielded the largest area under the curve (AUC = 0.92). **(B)** ROCs for differentiating poor (bad + questionable) data from good data. CNR and TCTV showed the best performance with AUC = 0.7–0.73. SNR, signal-to-noise ratio; SVNR, signal variance-to-noise ratio; CNR, contrast-to-noise ratio; CVNR, Contrast variance-to-noise ratio; TCTV, tissue contrast-to-tissue (intensity) variance; FWHM, full width-at-half maximum; CoM, center of mass.

In the visual inspection of 1569 sMRI data in the TRACK-TBI project, 1345 images (85.7%) were classified into ‘good,’ 199 (12.8%) into ‘questionable’ and 25 (1.5%) into ‘bad’ quality. The kappa statistic of the two raters (HT, RECB) was 92%, indicating excellent agreements between the raters using the protocol described in Supplementary Data 3. When merging the ‘questionable’ cases to the ‘good’ group, the auto QC for all QC metrics showed higher agreements with the visual inspection results compared to when merging the ‘questionable’ cases to the ‘bad’ group (0.61–0.91 vs. 0.51–0.73). The QC metric yielding the largest AUC was CNR (0.91 for good + questionable, 0.73 for bad + questionable), followed by CoM (0.88, 0.56), TCTV (0.87, 0.70), FWHM (0.85, 0.54), SVNR (0.74, 0.56), and CVNR (0.62, 0.51), respectively. At the best cutoff values, the auto QC of FWHM showed the highest classification accuracy, which was 0.86, followed by the analyses of CNR (0.84), CoM (0.84), TCTV (0.81), SNR (0.73), SVNR (0.70), and CVNR (0.58). The results including the cutoff values used for the best performance of the auto QC are summarized in Table 9 and Figure 6. We found that 3 or more QC metrics with ‘bad’ flags could be used to identify an image as ‘bad’ and result in the best agreement with the visual inspection (sensitivity = 85%, specificity = 87%, accuracy = 89%; overall AUC = 0.93). This was 3, 0, and 2% higher in sensitivity, specificity, and accuracy compared to the results using the CNR only that yielded the best result among all the QC metrics.

**TABLE 9:** ROC analysis QC metrics for fMRI.

		**Bad vs. Good and**	**Bad and Questionable**
		**Questionable**	**vs. Good**
*SNR*	AUC	0.7235	0.6732
	Sensitivity/Specificity	0.7090/0.7500	0.6465/0.6547
	Cutoff (*Z*-score)	13.0972 (−2.5500)	14.2491 (−2.3500)
*SVNR*	AUC	0.7369	0.5596
	Sensitivity/Specificity	0.6870/0.7083	0.5685/0.4798
	Cutoff (*Z*-score)	57.0564 (−1.6500)	77.4662 (−1.5000)
*CNR*	AUC	0.9189	0.728
	Sensitivity/Specificity	0.8171/0.8696	0.6744/0.6528
	Cutoff (*Z*-score)	5.6558 (−0.9500)	9.5552 (−0.2000)
*CVNR*	AUC	0.6169	0.5131
	Sensitivity/Specificity	0.6047/0.5652	0.4465/0.5648
	Cutoff (*Z*-score)	106.2423 (−0.400)	228.6781 (−0.300)
*TCTV*	AUC	0.8689	0.696
	Sensitivity/Specificity	0.7999/0.8261	0.5444/0.7963
	Cutoff (*Z*-score)	0.4680 (−1.1000)	1.3580 (0.2500)
*FWHM*	AUC	0.8447	0.5346
	Sensitivity/Specificity	0.8935/0.8261	0.5667/0.4861
	Cutoff (*Z*-score)	14.3189 (1.3000)	9.9080 (−0.3500)
*CoM*	AUC	0.8774	0.5559
	Sensitivity/Specificity	0.9446/0.7391	0.7428/0.3843
	Cutoff (*Z*-score)	0.2877 (2.8500)	0.0615 (0)

*For each QC metric, the area under the ROC curve (AUC), as well as the sensitivity, specificity and cutoff value at the best performance are displayed. For an easier choice of the cutoff values, we provide the readers the cutoff values in the original metric and *z*-score. We tested two different classifications: (1) Bad vs. Good and Questionable categories; (2) Bad and Questionable vs. Good. We found that the classification of (1) is overall more accurate using the QC metrics used in our system.*

## Discussion

Here, we have introduced the LONI QC system, a web-based and expandable system which features a rigorous workflow for the review and assessment of multimodal MRI including sMRI, fMRI, and DTI as well as CT. We also detailed the features of the user-friendly GUI that facilitates user’s execution of data uploading, initiating new QC, executing Auto QC, setting parameters for QC, visualizing the resulting QC metrics, vectors and 3D maps, evaluating and revising the QC results, and submitting the final QC. All these functionalities are found in the LONI QC website^8^ through the GUI that interacts with the various menus, or panels that were explained in the previous sections. A newly added tutorial helps the users follow the testing with demo data^9^ (yellow ‘tutorial’ button on the top-left corner), which will potentially increase the accessibility of the current functionalities in the system.

In a thorough evaluation of the system using various sets of data acquired from a single scanner and multiple sites and we found a strong degree of similarity among the datasets as well as distinguishing the characteristics specific to each dataset. The QA metrics are generally reproducible both within as well as consistent across subjects. In addition, we found that some data specific properties would be useful to be added as potential covariates in the automated QC method. Notably, anatomical changes due to normal patterns of aging may need to be considered in the user’s analyses, especially for SNR and CNR metrics.

Here, we extensively evaluated the utility of the auto QC by analyzing sensitivity and specificity of the cutoff value per sMRI QC metric to the identification of ‘bad’ quality images in comparison to visual inspection. Our results can be used as a guideline for the proper settings for the QC process and as users’ interpretation on the QC in their own data. To the best of our knowledge, the LONI QC is the first online QC system that uniquely supports to perform the image QC of multi-contrast and multimodal brain imaging data. The LONI QC system provides users a various level of image QC from the first aid of the user’s own image quality assessment to the high-end QC that automatically flags bad quality images based on the user’s setting of cutoff values. This service provides various options of MRI QC (i.e., computation of QC metrics, auto QC, user’s own evaluation on QC metrics and visual QC), depending what type of the QC the users prefer to perform. LONI QC differs from the previously developed tools that push the QC to correction of bad quality images by de-noising or removing the voxels or volume frames affected with artifacts (Zhou et al., 2011; Li et al., 2013; Liu et al., 2015). These correction processes are computationally costly.

### Pros and Cons of LONI QC Compared to Other Extant QC Systems

Compared to previously developed QC tools, the current QC system has the following new features: It is the first completely online system which is supported by various web-browsers and requires no preinstalled software. The online system allows users to anonymously upload imaging data to the LONI QC system, either through LONI Integrated Data Archive (IDA) or using a direct uploading interface, thus having no issue of identity theft in the processed data. The automated QC has been set with the default parameters using those determined as in Table 9, which can be adapted to the user’s data. It computes a standard set of QC metrics that have been described in the literature and performs a standardized QC via an automated pre-processing system which is specifically designed to generate a range of scalar and vector statistics along with derived images. The QC data processing is performed on the LONI processing grid in the USC Mark and Mary Stevens Neuroimaging and Informatics Institute making possible parallel computing using a cluster of thousands of central processing units (CPUs) whereas the previously developed approaches were designed to work on a single-core of the personal computer where the source code was downloaded. LONI QC system also features a user-friendly web-based GUI and a tutorial with demo data that help particularly novice users get familiar with the QC system.

There are several important considerations that potentially improve the LONI QC approach compared to the current limitations of other approaches: First, it is freely accessible through the Internet so that it is impossible to process offline data while also provided as a downloadable framework which runs on the user’s local computing environment – but which does necessitate the independent installation of prerequisite software. The LONI QC system is partly dependent on the data archiving capacity of the IDA. Large size image datasets are preferably collected and archived in the IDA prior to the QC execution. The direct data uploading module has been tested with a small set of data (*n* < 30 at one uploading) with a small number of simultaneous network connections (number of users < 5). This eventually prevents the users from keeping their image data in our online storage after QC reports are generated. The capacity of the network traffic and the data storage in our computing cluster when using the direct uploading module is currently being expanded and tested by our developer team, allowing the affordability of more users who have difficulty or are reluctant to access LONI QC system through the IDA. Second, the current system has yet to support the auto QC of DTI data as no scalar QC metrics for DTI are computed. Roalf and his colleagues in their recent work (Roalf et al., 2016) devised a number of DTI QC metrics and showed a high degree of sensitivity and specificity. Indeed, it is particularly challenging for a human rater to assess the quality of the time series volumes of fMRI and the multidirectional volumes of DTI data. Therefore, we plan to include the quantitative metrics discussed by Roalf et al. (2016) or equivalent ones, to support the auto QC of DTI data in future releases of LONI QC. Third, the optimal setting of cutoff values for auto QC may vary depending on the image sequence and weighting methods, as also shown in the current study. In pediatric imaging data, a greater degree of motion artifact can be involved compared to adult data. This may require an adaptive setting regarding such confounding effects. The current version of the system provides the default setting with the parameters achieved in our evaluation (see Table 9) with a flexibility of scaling cutoff values by the users. Furthermore, the current system only analyzes each QC metric separately using a univariate fashion. A machine learning approach using multivariate modeling of the QC metric’s distribution can classify the quality of each image data with a higher accuracy as found in Pizarro et al. (2016), Esteban et al. (2017), and Fonov et al. (2018). Fourth, recent studies (Li et al., 2013; Oguz et al., 2014; Power et al., 2014) developed and evaluated methods to reduce, correct or remove some types of artifacts existing on DTI and fMRI images. Such image reconstruction or enhancement, albeit with the possibility of inducing a bias, may help to decrease the chance of permanent exclusions of the cases with a bad image quality from the subsequent biological or clinical analyses. Fifth, a previous study (Mortamet et al., 2009) designed QC metrics that are sensitive to the identification of machine-inherent noises (e.g., Gaussian noise, aliasing, zipper pattern) by masking out the head area in measurement whereas we included a more variety of QC metrics that can capture the types of noise occurring inside (e.g., head motion) and outside the brain region. Finally, a future improvement of the study is to evaluate the effects of running LONI-QC on the performance in subsequent image analysis. This can be hinted by the attempts made for the quality assurance of post-image processing such as in the studies evaluating brain structural segmentation on sMRI (Keshavan et al., 2017) and fiber tractography extracted from DTI data (Sommer et al., 2017).

### Reproducibility of the QC Metrics Adopted in LONI QC System

The choice of metrics when evaluating the quality of a neuroimaging dataset has substantial implications for how data processing steps are carried out subsequent to image acquisition. In the current system, we included a broad range of QC metrics modeling various aspects of the image artifacts possibly occurring during image acquisition. Many of these metrics were also chosen or developed by other studies in the literature (Friedman and Glover, 2006; Power et al., 2012; Li et al., 2013; Marcus et al., 2013; Pizarro et al., 2016). The histogram analysis of these metrics showed their reproducibility in multiple datasets including those acquired with a single setting of imaging acquisition parameters or with multiple settings of imaging parameters used in multiple scanners. The distributions of these metrics were not significantly influenced by different parameter settings if the analyzed images were acquired using the same sequence (e.g., T1-weighted) and the same modality (sMRI, fMRI, DTI). On the other hand, results in the analysis of T1-weighted sMRI suggest that the means of SNR and CNR can differ when imaging elderly or a dementia patient populations. In the analysis of the possible introduction of larger motion artifacts in younger subjects, we did not observe the influence of the age variation on the QC metrics measured in the data tested here. While this finding shows the age would not be a confounding factor in younger adult cohorts of TRACK-TBI, it does not necessarily imply that the severity of motion artifacts in pediatric data is as same as that in adult data. Previous studies indeed showed that some obvious bad quality images displayed a significant correlation between QC metrics and age (Roalf et al., 2016) and prospective motion correction improved diagnostic sensitivity in pediatric data (Kuperman et al., 2011).

When data are collected in a single machine with uniform imaging parameters, the variance of the QC metrics becomes significantly smaller, suggesting that the variance in the multi-site data partly explains the machine characteristics and the difference in imaging parameters. On the other hand, differences in the image sequence (e.g., T1-weighted, T2-weighted, FLAIR), even acquiring a same modality image appear to create a significant difference in their distribution, suggesting that the direct comparison of the QC metrics resulting from two datasets acquired using different image sequences may not be suitable. The users may need to consider the aforementioned factors in setting the proper cutoff values in the auto QC to identify bad quality images.

Correlational analyses illustrated that the major proportion of the QC metrics in sMRI were not associated each other whereas many in fMRI showed significant correlations each other. The main reason why the many fMRI QC metrics were correlated is likely that these metrics characterize temporal signal changes or head displacements that can be driven by head motion. The LONI QC system was designed with this in mind, and one of its strengths is that it calculates for the users not only standard—and occasionally correlated—metrics such as the CNR and CVNR, but also more information-rich evaluations. In doing so, the LONI QC system provides a platform for evaluating the relationships between a wide variety of QC metrics and allows the users to choose those metrics which may be more relevant in their studies. Generating QC vectors and 3D maps, a greater variety of choices is given for the users to perform image quality assurance and control in depth. This idea is not different from those adopted in the previously published works (Oguz et al., 2014; Esteban et al., 2017). Eventually feature reconstruction approaches such as principal component analysis (Tenenbaum et al., 2000) or independent component analysis (Cao et al., 2003) may reduce the number of QC metrics while keeping their QC performance by projecting them on to the axes that explain larger variations of the data or better explain the information implied in the data.

### Auto QC: Comparison to Visual Inspection

Recent studies have made an unprecedented effort to acquire an enormous size of MRI dataset in line with the emergence of the new generation of the analysis in ‘BIG’ data. Nearly every week, more than 1000 new scans of sMRI, fMRI or DTI data are archived in the repository of the LONI-IDA. The tedious and time-consuming visual inspection in the quality of such massive datasets is not practical. Automated QC that quantifies image QC metrics, and labels the degree of image quality is of major interest and there have been recent attempts to substitute the manual QC procedure. In the current paper, we introduced such an automated procedure that used various QC metrics and their cutoff values to flag bad quality images. The strength of LONI QC and other similar methods that were proposed recently (Oguz et al., 2014; Pizarro et al., 2016; Roalf et al., 2016; Esteban et al., 2017) lies on the use of multiple QC metrics that characterize various aspects of image artifacts involved in the brain image acquisition. Furthermore, these metrics have an ability to differentiate the degree of the artifact severity as they are continuous and not categorical or dichotomous (i.e., good or bad).

However, the results from automated QC and similarly those previously published (Pizarro et al., 2016; Roalf et al., 2016; Esteban et al., 2017) do not always fully agree with the visual inspection results. This is because the univariate analysis of each metric may be able to detect one type of the image artifact whereas the visual assessment performs a comprehensive evaluation where the deterioration in image quality is multifaceted with simultaneously occurring multiple noise types. The use of thresholds along with the number of simultaneously occurring ‘bad’ QC metrics further improved the classification accuracy. Another study (Pizarro et al., 2016) used a multivariate analysis by employing a support-vector machine-based classifier and showed the potential improvement against univariate analyses. Interestingly, the QC metrics utilized in LONI QC were more sensitive to the classification when merging the ‘questionable’ or ‘moderate’ quality images to ‘good’ images. We separately performed the 3-class classification, but this showed a worse result (AUC = 0.5–0.6) than 2 class classification. This suggests that questionable cases would not be clustered as an independent "moderate" group, but their characteristics would be closer to that of the "good" group. However, it is not clear whether or not the questionable quality images are potentially problematic in the post-image processing or the subsequent biological/clinical analyses. Further examination of quality clustering will form the basis of ongoing activities for the LONI QC framework.

## Conclusion

Quality control of neuroimaging data is an essential, though a complex and challenging component of image processing and analysis. Although many previous studies have aimed to identify an ideal set of measures which can distinguish between images of good and bad quality, it remains the case that different researchers have different intuitive, qualitative and quantitative standards of what image quality should be, and of how that quality ought to be quantified. The LONI QC system was specifically designed with these considerations in mind, and is the first both web-based and freely-available QC system which provides users with the ability to specify their own standard of image quality, automatically apply that standard to their data, and then download the results of their QC analysis in CSV and/or PDF format for further post-processing using the tools and methods of their choice. Because it accommodates a wide variety of imaging modalities, the LONI QC system can appeal to a substantial cross-section of researchers in the neuroimaging community who are interested in applying and maintaining the highest standards of image quality to their image analyses and, by extension, to their research efforts. The streamlined integration of the LONI QC system with the LONI IDA and with the LONI Pipeline—both of which are widely used by neuroimaging researchers—throws additional weight behind the argument that this novel, state-of-the-art system can be easily adopted by a large number of neuroimaging researchers worldwide, thereby potentially leading to the formulation and adoption of a much-needed standardized protocol for neuroimaging QC and analysis.

## Author Contributions

HK: study design, evaluation, statistical analysis, and manuscript drafting. AI: study design, evaluation, and manuscript drafting. SH, PP, and MP: implementation and system maintenance. HT: assisted in evaluation. RE: manual/visual quality control. BD, S-LL, KC, and ML: statistical analysis and manuscript editing. LZ: data acquisition, statistical analysis, and manuscript editing. KLC: data accessing and implementation. PM and GM: data acquisition and sharing. JVH: study design, manuscript editing, and revising. AT: study design, primary funding, and manuscript editing.

## Conflict of Interest Statement

The authors declare that the research was conducted in the absence of any commercial or financial relationships that could be construed as a potential conflict of interest.

## Footnotes

1 http://preprocessed-connectomes-project.org/quality-assessment-protocol/

2 https://mriqc.readthedocs.io/en/stable/

3 http://pipeline.loni.usc.edu

4 http://ida.loni.usc.edu

5 https://fsl.fmrib.ox.ac.uk/fsl/fslwiki/BET

6 http://www.fil.ion.ucl.ac.uk/spm/

7 http://www.trackvis.org

8 http://qc.loni.usc.edu

9 https://qc.loni.usc.edu/dashboard
