# Supplementary material for: The LONI QC System: A Semi-Automated, Web-Based and Freely-Available Environment for the Comprehensive Quality Control of Neuroimaging Data
Source: Front Neuroinform. 2019 Aug 28;13:60. doi: 10.3389/fninf.2019.00060 (PMC6722229; doi:10.3389/fninf.2019.00060)
Supplement: Supplementary file 1 [file Data_Sheet_1.docx]

| **Name of Tool / Reference** | **Modalities** | **Description** | **Datasets Analyzed** | **Findings and performance of classifiers** |
| --- | --- | --- | --- | --- |
| DTIPrep tool, Oguz et al. Front Neuroinform. 2014, Liu et al. Proc SPIE Int Soc Opt Eng. 2010 | DTI | Number of QC metrics: 1. Type of QC: auto. GUI: yes. Prerequisite software: None. Publicly available: yes. QC method: univariate (Entropy-based measurement of the principal diffusion direction distributions) | IBIS (n=400+, infants) | Entropy based measurement was able to automatically detect 100% of the severe and 67% of the milder vibration artifacts. |
| Roalf et al. Neuroimage. 2016 | DTI | Number of QC metrics: 4. Type of QC: auto. GUI: no. Prerequisite software: FSL, AFNI. Publicly available: yes. QC method: univariate (TSNR) | PNI (n=1500+, healthy youths) | Temporal SNR (TSNR) best differentiated poor data from excellent data and maximum voxel intensity outlier count differentiated good from excellent DTI data. TSNR was able to identify good from poor data with a sensitivity of 85% and a specificity of 84%. |
| Lauzon et al. PLoS One. 2013 | DTI | Number of QC metrics: 4. Type of QC: manual. GUI: yes. Prerequisite software: MATLAB. Publicly available: yes. QC method: univariate | Own data (n=607, healthy subjects all ages) | Using the QC report generated using post-processed DTI data significantly reduced errors that can be introduced by a novice's evaluation of data quality. |
| Power et al. Neuroimage. 2014 | fMRI | Number of QC metrics: 5. Type of QC: auto. GUI: n/a. Prerequisite software: n/a. Publicly available: no. QC method: univariate + within subject regression | Own data (n=80, healthy adults) | Found that motion-related signal changes can persist over multiple fMRI time points (>10 s) and that global signal regression combined with ‘scrubbing’ eliminated significant differences in functional connectivity between groups of high and low motion adults. |
| Christodoulou et al. Magn Reson Imaging. 2013 | fMRI | Number of QC metrics: 6. Type of QC: auto. GUI: n/a. Prerequisite software: n/a. Publicly available: no. QC method: multivariate (Clustering, nearest-neighbor) | Own data (n=45, healthy adolescents) | Features derived from the mean square derivative of the signal before and after realignment was able to classify all motion corrupted datasets correctly using leave-one-out CV. |
| Connectome Workbench, Marcus et al. Neuroimage. 2013 | sMRI, fMRI | Number of QC metrics: 9. Type of QC: manual. GUI: yes. Prerequisite software: no. Publicly available: yes. QC method: manual evaluation | HCP (n=126, healthy adults) | Put in place QC procedures for the HCP and developed a method for cross-modality data visualization and exploration. |
| PCP Quality Assessment Protocol Shehzad et al. Front Neurosci. 2015 | sMRI, fMRI | Number of QC metrics: 17. Type of QC: manual. GUI: yes. Prerequisite software: AFNI, Nipype. Publicly available: yes. QC method: manual evaluation | ABIDE (n=1113, healthy + ASD subjects), CoRR (n=1400+, multiple datasets) | The percentage of artifactual voxels outside the brain and the SNR were the best predictors of structural data quality scores. EFC, FWHM, Percent FD, and GSR were all significant predictors of functional data quality. |
| MRIQC, Esteban et al. PLoS One. 2017 | sMRI | Number of QC metrics: 56. Type of QC: auto. GUI: yes. Prerequisite software: FSL, AFNI, ANTs, Nipype. Publicly available: yes. QC method: multivariate (Binary classification using random forests) | ABIDE (n=1102, healthy + ASD subjects) | Achieved 89% CV accuracy with the coefficient of joint variation as the most important feature. |
| Mortamet et al. Magn Reson Med. 2009 | sMRI | Number of QC metrics: 2. Type of QC: auto. GUI: n/a. Prerequisite software: n/a. Publicly available: no. QC method: univariate | ADNI (n=749, healthy + MCI + AD) | The two quality indices based on: (i) detection of artifactual voxels using thresholding and morphological operations and (ii) noise distribution analysis were able to discriminate poor from good quality data with a sensitivity and specificity of >85%. |
| Gedamu et al. J Magn Reson Imaging*.* 2008 | sMRI | Number of QC metrics: 7. Type of QC: auto. GUI: n/a. Prerequisite software: n/a. Publicly available: no. QC method: univariate | Own data (n=45-250) | Detected ghosting artifacts with a 95% sensitivity and 96% specificity, as well as verification of MRI parameters and gadolinium enhancement with 100% accuracy. |
| Woodard and Carley-Spencer. Neuroinformatics. 2006 | sMRI | Number of QC metrics: 239. Type of QC: n/a. GUI: n/a, Prerequisite software: n/a. Publicly available: no. QC method: univariate | MNI-ICBM (n=143, healthy adults) | Wavelet decomposition-based measures most significantly discriminated the artifactual images from good quality images |
| Pizarro et al. Front Neuroinform. 2016 | sMRI | Number of QC metrics: 6. Type of QC: auto. GUI: n/a. Prerequisite software: n/a. Publicly available: no. QC method: multivariate (Support Vector Machines) | Clinical Brain Disorders Branch CBDB Sibling Study, NIH Clinical research volunteer program (n=1457, healthy subjects + neuropsychiatric patients) | Achieved 80% accuracy in discriminating poor from good quality data (10-fold CV). Artifact specific features are the most powerful features and these regional features could also be used for localizing artifacts. |
| Alfaro-Almagro et al. Organization for Human Brain Mapping.  2017 | sMRI | Number of QC metrics: 190. Type of QC: auto. GUI: n/a. Prerequisite software: FSL. Publicly available: yes. QC method: multivariate (Bayesian networks + naïve Bayes combined) | UK Biobank (n=10000+, healthy adults) | Achieved 87% accuracy (10-fold CV) in classifying usable from unusable datasets. |
| Glover et al., J. Magn. Reson. Imaging. 2012 | fMRI | Based on the Function Biomedical Informatics Research Network (FBIRN): manual protocol, GUI: n/a | N/A | Provided practical components for the QC, design and execution of Multi-Center fMRI studies. |
| Rosen et al., Neuroimage. 2018 | sMRI | Number of QC metrics: 7. Type of QC: auto. GUI: n/a. Prerequisite software: QAP version 1.0.3 & FreeSurfer. Publicly available: no. QC method: univariate | PNC (n=1598, healthy adolescents), independent dataset (n=242, healthy adults) | Achieved 50-86% AUC for QAP metrics and 98-99% AUC for FreeSurfer Euler number (metric of surface topology error) |

Supplementary Table 1: Literature review of studies which performed systematic evaluations of QC methods for DTI, fMRI and sMRI.

Abbreviations: ABIDE: Autism Brain Imaging Data Exchange Collection, ASD: Autism Spectrum Disorder, CoRR: Consortium for Reliability and Reproducibility, CV: cross-validation, EFC: Entopy Focus Criterion, FD: Framewise Displacement, FWHM: Full-width at Half Maximum (measure of smoothness), GSR: Ghost-to-signal-ratio, HCP: Human Connectome Project, IBIS: Infant Brain Imaging Study, MNI-ICBM: Montreal Neurological Institute - International Consortium of Brain Mapping, PCP: Processed Connectomes Project, PNI: Philadelphia Neurodevelopmental Cohort. AUC: area under the curve
