# Supplementary material for: The LONI QC System: A Semi-Automated, Web-Based and Freely-Available Environment for the Comprehensive Quality Control of Neuroimaging Data
Source: Front Neuroinform. 2019 Aug 28;13:60. doi: 10.3389/fninf.2019.00060 (PMC6722229; doi:10.3389/fninf.2019.00060)
Supplement: Supplementary file 2 [file Data_Sheet_2.docx]

*The following information is provided to facilitate the user’s accessibility to the online LONI QC system. This includes details of the processes (e.g., software or tool names used) that were not explained in the main text as well as how to use the GUI of the current system to use each of the functions implemented in the system. The section numbers correspond to those in the Materials and Methods of the main text.*

*1. Initialization (Figure 1-1)*

*1.1. Creating an online account and starting the LONI QC system*. The first step towards running the system for users is creating their account at the welcome page (<https://qc.loni.usc.edu>**; Figure 2a**). Clicking the
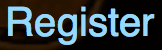
 button on the right-hand side will open a subscription page that asks users to fill out basic information. The created account can exchangeably be used for the access to the LONI Image Data Archive (IDA; <http://ida.loni.usc.edu)>, which eases data-uploading through the IDA. Once the account has been successfully created, each user can log-in the QC system and access the main page where the user can find a demo image data collection including a few individual images of each modality e.g. sMRI, fMRI and DTI on the left panel in order to get familiar with the system. The main page consists of three separate panels (**Figure 2b**): The right one provides various menus that are used to select the existing data collections (groups of scans created either by using the IDA or by uploading the user’s own data), upload a local collection of image data (user’s own data). The collections from IDA should be linked with the user to appear on the panel (details are found in <http://ida.loni.usc.edu> . Once the user selects a collection in the left panel, all the image data included in this collection panel appears on the middle panel. The user can select an image ID or multiple IDs on the panel. Finally, on the right panel, the user can initialize a new QC by clicking
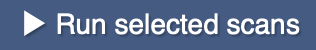
, check and evaluate the QA results (
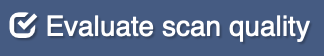
), generate the summary report of the resulting QC (
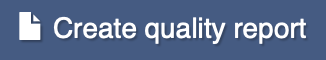
), download the reports in a csv file (
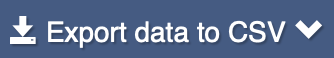
), refresh the status of QC in the selected collection (
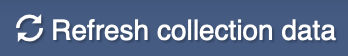
) or setting the auto QC cutoff ranges (
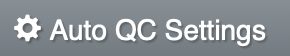
). The user can view the overall distribution of each QA metric for the dataset of the selected collection by clicking the menu button,
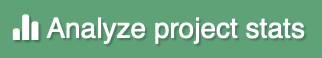
.

*1.2. Uploading data.* To enable the submission of an image data by users, the LONI QC system either interacts with the LONI-IDA or uses a separate module that allows the users to directly upload their data to the QC system. Uploading data through either the IDA or the direct upload module is permitted using DICOM or nifty format, allowing collection of full header information including scanner protocol parameters. An engine for flexible data de-identification and encrypted file transmission helps to ensure compliance with patient-privacy regulations. The users can upload their own data to the QC pipeline by selecting the
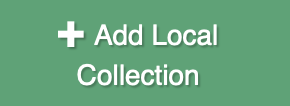
 button on the left panel. Clicking this button will display a module on the main panel, enabling the users to select a folder containing DICOM files related to an image, to select multiple folders simultaneously, or to select multiple NiFTI format files. The uploaded images will be included in the ‘local’ data collection (shown in the collections window in the right panel once the upload become successful) or in an existing or a new collection that the users have selected or created. Uploading data through the IDA automatically archives the data in the IDA securely, which requires no specialized hardware, software or personnel. The IDA and the direct upload module automatically extract relevant metadata from all de-identified image files. If the file was uploaded on to the IDA, integration of the LONI Debabeler file format translation engine allows users to download image data in a number of file formats including NiFTI, minc, mgz, nrrd and etc. in addition to the original file format. All data are stored on supplemental servers with daily and weekly on- and off-site backups.

*2. Computation of QA metrics for various modality images (Figure 1-2)*

Selecting the
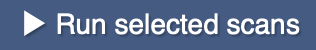
 button (right panel; Figure 2b), the users can submit image data from the selected data collection to the QC pipeline. This action initiates the system for computation of the QA metrics.

*2.1. Workflow and QA metrics for sMRI and CT*. In the first step, the Java module IDAGet downloads DICOM data for each subject from the LONI IDA. In the second step, the freely available dcm2nii program (<https://www.nitrc.org/projects/dcm2nii/>) is used to convert DICOM files to the NIfTI format. DICOM and NIfTI header information—including XML metadata—is then extracted using the MRIConvert program available in FreeSurfer (<http://freesurfer.net/>), the dicom_hdr program of AFNI (<https://afni.nimh.nih.gov/afni/>) and the DICOM Summarize program (<https://wiki.loni.usc.edu/index.php/DICOM_Summarize>).

*2.2. Workflow and metrics for DTI*. The workflow for processing DTI data is similar to that for sMRI, with the notable exception that additional checks are performed to ensure that data for all gradient directions are available. If this is not the case, the workflow generates error messages to the user. The python script NIfTIQA, a part of tools distributed by The Biomedical Informatics Research Network (BIRN), is used to mask the head in each slice, and generate a histogram of image intensities and compute descriptive statistics for each volume. A detailed report containing information about the number of voxels, mean intensity, standard deviation, SNR, and minimum and maximum intensities for each slice is also generated using this tool. The BIRN fMRIQA_GENERATE tool and the DTI Recon tool in TrackVis (<http://trackvis.org>) are then used to calculate the DTI QA metrics listed in the main text. The DTI_tracker and Spline Filter tools are used to create tractography streamlines, which are displayed in the LONI Image Viewer.

*2.3. Workflow and metrics for fMRI*. Tools from the FSL package and from AFNI are used to calculate the metrics listed in the main text.

*2.4. Workflow and metrics for phantoms*. The QC protocol for phantoms is similar to that for each type of imaging (sMRI/CT, DTI or fMRI, with minor differences. For fMRI phantoms, the BIRN fMRIQA_PHANTOMQA Python script is used instead of the fMRIQA_PHANTOMQA to calculate the QA metrics.

*2.5. User’s Access to the results from the LONI QC process (Figure 2b).* Once a QC job is submitted using the
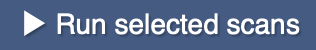
 button, the users can view the QC status of each image on the middle panel. The LONI QC system assigns each scan to one of five status categories dependent upon the completion of either the system’s QA metric computation or the user’s QC, which is displayed on the right-bottom panel of the LONI QC website. These five categories are:

- *processing*: volumes submitted to the QC system which have not yet been processed
- *ready to evaluate*: volumes processed by the QC system which are to be reviewed and labeled (good, acceptable or bad) by the user
- *evaluating*: volumes for which user evaluation is ongoing and as yet incomplete
- *finalized*: volumes for which the user evaluation has been completed
- *invalid data*: volumes whose format or DICOM header information is incompatible with the universal DICOM standard and therefore with the QC system

On the middle panel, the system also displays a list of the QC information of the image being evaluated, including:

- *image ID*: a unique numeric identifier which is assigned by the QC system to each volume
- *modality*: the type of MRI scan (e.g. sMRI, DTI, or fMRI)
- *project*: the project name to which each image belongs (e.g., ADNI, ICBM).
- *patient ID*: this numeric field is used to identify the patient from whom the data were acquired while preserving patient anonymity and HIPPAA compliance
- *site key*: for multi-site studies, this numeric code identifies the site where the volume was acquired
- *scan date*: the date that the data were acquired
- *research group*: the type of study to which the data belongs (e.g. MRI)
- *Status: one of 5 QC status categories as explained above*
- *Select*: to *link* the selected image(s) with the actions listed on the right panel.

*3. Image QC (Figure 1-3; Figure 2d)*

*3.1. User’s qualitative and quantitative QC of image data*. As described above, the QC status for each scan data changes to ‘awaiting evaluation’ once the image data were processed and QA metrics have been computed. In the right-bottom main panel, the graphical user interface (GUI) of LONI QC pipeline then is used for users’ visual inspection of the quality of images as well as their quantitative evaluation of QA metrics (**Figure 2d**).

- Quantitative evaluation: To evaluate each scalar QA metric, the GUI provides a horizontal box plot where the users can find the QA metric value of the currently examined image and the box (in blue) whose size indicates the SD of the given metric in the selected data collection or the project (**Figure 2d-1**). Thus, the users can evaluate whether the currently measured QA metric stands inside or outside the range of the values measured for the whole dataset to analyze. To evaluate each vector metric, the GUI provides a plot (**Figure 2d-2**) to visualize the profiles of the given vector metric along the image slices (sMRI), time series volumes (fMRI) or gradient directional volumes (DTI). 3D maps of QA are displayed with all the slice images in the direction of the image acquisition (**Figure 2d-3**).
- Visual inspection: The GUI is fully integrated with the LONI Viewer (**Figure 2d-4,5**), which is a sophisticated, web-enabled neuroimage viewing system. The viewer is available both at the online LONI QC system and as a stand-alone application which can be downloaded from the LONI website (<http://pipeline.loni.usc.edu/products-services/brain-viewer>). When viewing DTI tractography files using the LONI Viewer, interactive features are available to threshold WM tractography bundles according to their lengths, to perform three-dimensional (3D) rotations and to create animations. However, the webgl library for the 3D image viewer may not be fully compatible with Safari web browser and may slowdown or crash the operating system.

*3.2. Auto QC*. The autoQC setting menu
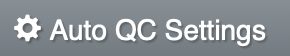
 is found on the right-bottom panel. By clicking
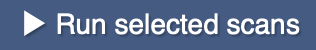
, AutoQC is activated along with computation of QA metrics: The system’s GUI provides the users a way to set a range for each QA metric, with which they can classify the resulting metric to the ‘good’, ‘questionable’ or ‘bad’ category. Our system also displays the location of each metric value relative to its user-defined range. Selecting the
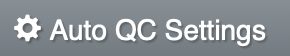
, the users can enter the auto QC setting page where they can change these cutoff ranges and also find the mean (μ) ± 1 SD (σ), 2 σ and 3 σ of each QA metric computed over the entire set of datasets that the QC system has processed. These normalized statistics are then used to help the users choose the cutoff range (**Figure 2c**). For example, the range within which a metric can be defined as ‘good’ could be set as μ ± 2σ, or in a similar way. Metrics whose values fall outside this interval could be then be labeled automatically as ‘poor’. If a user does not wish to set up the cutoff thresholds in the setting page then system automatically sets them at the locations of μ ± 2σ. These mean and SD are determined based on a choice of numerous different distributions obtained using: 1) Protocol compliance: The entire dataset that has been processed in the QC system; and 2) Computed metrics: the dataset that were evaluated in the current paper representing each specific image sequence (i.e., sMRI, fMRI, DTI, CT).

Once the Auto QC settings have been specified and saved, users will find that the QC report page includes automatic assignment of a QC label for each metric. It is noted that the QA metrics are set to ‘questionable’ only when they fall into the range that the users have separately specified as ‘questionable’ (in orange color box). Finally, if more than a user-specified number (system default: 3) of computed metrics are flagged as ‘bad, the system flags the assessed case as ‘bad’ and suggests it to be more closely checked.

To finalize a QC report review and submission, the user is requested to provide an overall evaluation of the volume on the basis of the result of the auto QC as well as that of their own qualitative QC (**Figure 2e**). The GUI of the system also provides the user a way to enter pertinent, useful or necessary comments in a text box provided for this purpose. The system allows up to two users to specify the date when the QC report was finalized, as well as the name or initials of the user who provided the evaluation. Once reviewed, the report can be either only saved, or saved and submitted to the QC database. If the report is only saved, ulterior changes can still be made until its submission. In each case, the
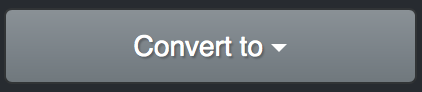
 menu button located in the bottom of the QC report page is provided for users to convert the report into either xML, PDF or CSV format for further distribution or analysis (**Figure 2e**). The entire QC process is estimated to be completed within less than a minute for each scan by an expert neuroimaging researcher who has been trained on the usage of the system.

*3.3. QC study summaries*. This feature is found as the button
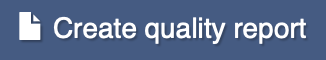
 on the right panel and allows the user to consolidate QC results of the images selected on the middle panel into overall reports of data quality and to monitor how this quality varies across the sites involved in a study, across modalities or as a function of time.
