# Supplementary material for: The LONI QC System: A Semi-Automated, Web-Based and Freely-Available Environment for the Comprehensive Quality Control of Neuroimaging Data
Source: Front Neuroinform. 2019 Aug 28;13:60. doi: 10.3389/fninf.2019.00060 (PMC6722229; doi:10.3389/fninf.2019.00060)
Supplement: Supplementary file 3 [file Data_Sheet_3.docx]

# **Image Artifacts on sMRI**

**Motion/ Ringing**

Mild Moderate Severe


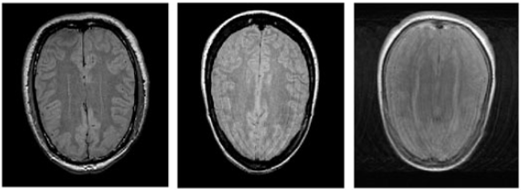


Subject motion during acquisition causes ghosting and blurring in the image. Blurring follows the motion direction, and corresponds to the distance moved. A truncation artifact appearing in the frequency encoding direction can be differentiated from a modern artifact by the absence of image blurring and the regular increase (Zhuo & Gullapalli, RSNA 2006).

**Flow/ Zipper**

Mild Moderate Severe


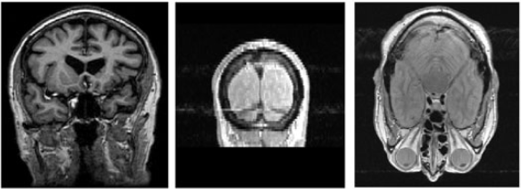


Blood flow is a type of motion-related artifact that appears in the phase encoding direction. The zipper artifact is caused by the electromagnetic interference inside the magnet room. It presents as noise in the frequency encoding direction (Zhuo & Gullapalli, RSNA 2006).

**Inhomogeneity**

Mild Moderate Severe


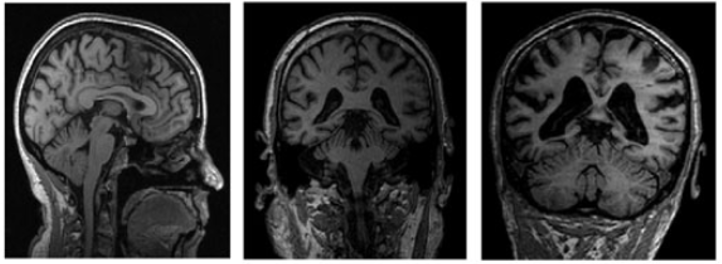


Image intensity inhomogeneity appears as a change of intensity across the brain. Possible causes are elements such as lack of coil uniformity, inadequate radio frequency or external radio frequencies, patient position and anatomy (Condon *et al* 1987, Simmons *et al* 1994)

**SNR (Signal to Noise Ratio)**

High Low


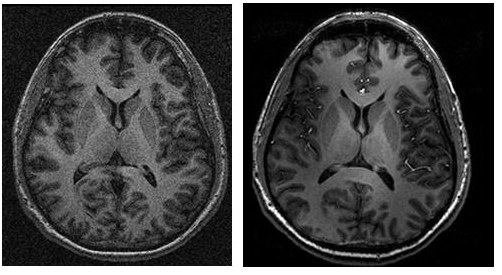


Signal to noise ratio (SNR) is a generic term, which in radiology is measure of how much true signal (e.g. reflecting actual anatomy) versus how much noise (e.g. random quantum mottle) a particular image has, which results in a grainy appearance (Dr Henry Knipe and Dr J Yeung *et al*).

**Magnetic Susceptibility**


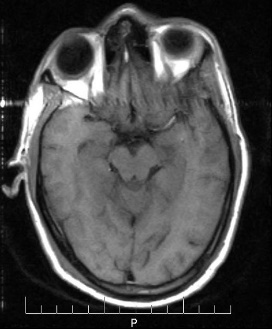

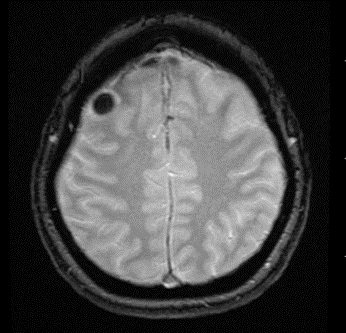

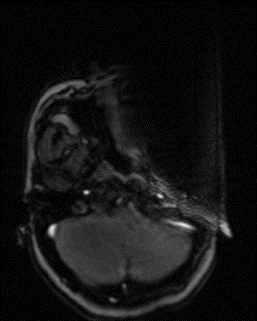


**Magnetic susceptibility artifact** refers to a distortion in the MR image especially seen while imaging metallic orthopedic hardware or dental work. This results from local magnetic field inhomogeneities introduced by the metallic object into the otherwise homogeneous external magnetic field B0 (Dr Bruno Di Muzio and Dr Usman Bashir *et al*).

**Wrap/ Aliasing**

Does not touch skull Touches Skull Penetrates brain


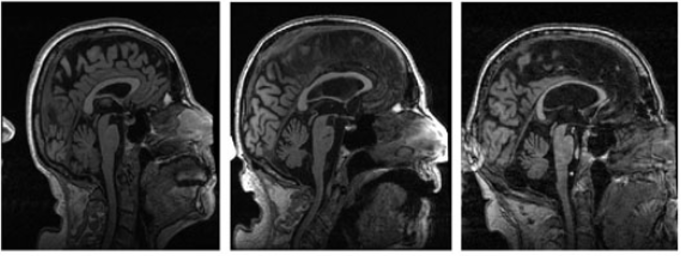


Wrap around artifacts happen when the size of the imaged object is larger than the field of view. Filters in the frequency encoding direction can remove the artifact (Pusey *et al,* 1986). If the wrap distorts the brain, the image should fail QC.

**Head Coverage**

Clips skull Clips brain


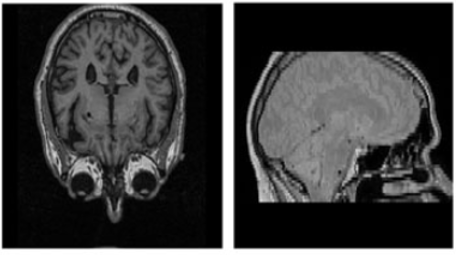
Head Coverage artifact appears when the border of an image occludes either the skull or the brain. If the brain is still fully visible, the image may still pass QC.

**Common persistent artifacts in EPI-based FMRI and DTI**

*1. Aliasing, or wraparound (described previously in sMRI)*

Aliasing effects in the frequency and phase encoding dimensions.

*2. Gibbs artifact, or ringing (described previously in sMRI)*

The origin of the ringing problem and demonstrations in phantom and brain data.

*3. Abnormally high N/2 ghosts (1/2)*

Subject-dependent conditions:

• Asymmetric orientation of the subject's head leading to a poor shim

• Poor shim as a result of subject motion during/immediately after shimming

• Presence of FOD or an implant causing a poor shim

Scanner-dependent conditions:

• Rotated read/phase encode axes

• No fat suppression

• Mechanical resonances

• Excessive ramp sampling

*4. Distortion and dropout*

fMRI / DTI data are mostly collected with Echo Planar Imaging (EPI), which suffers great geometric distortion (stretch, compress, or shear) and dropout due to field inhomogeneity induced by the susceptibility difference of different tissues (Hutton et al. NeuroImage 16, 2002) Distortion can also cause the inaccuracy of fiber tracking. An Example is shown below:


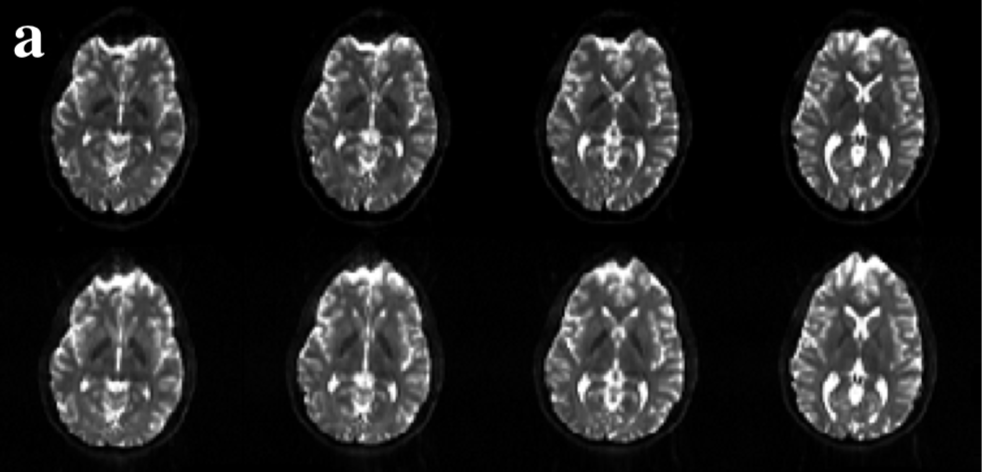

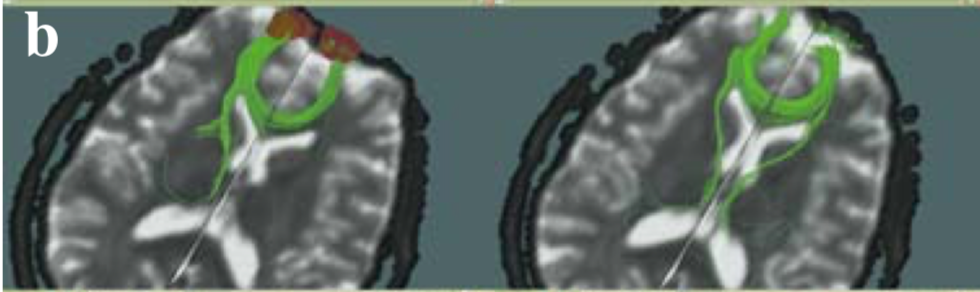


5. RF interference

RF screening, adding devices to the scanner environment, modifying the magnet room, and standard operating procedures for fMRI labs.

6. Receive coil heterogeneity

Receive fields for phased-array RF coils, and removing receive field heterogeneity with prescan normalization.

7. Subject movement

• Eye movements

• Head nodding

• Talking

• Coughing, swallowing, yawning and sneezing

• Body movements

8. Signal drift due to gradient heating

Use of the scanner in a non-steady state.

**Rare intermittent FMRI / DTI artifacts**

Spiking, sparking and arcing

**(main reference:** [**https://practicalfmri.blogspot.com/2012/09/understanding-fmri-artifacts-contents.html**](https://practicalfmri.blogspot.com/2012/09/understanding-fmri-artifacts-contents.html)**)**
