# Supplementary material for: The LONI QC System: A Semi-Automated, Web-Based and Freely-Available Environment for the Comprehensive Quality Control of Neuroimaging Data
Source: Front Neuroinform. 2019 Aug 28;13:60. doi: 10.3389/fninf.2019.00060 (PMC6722229; doi:10.3389/fninf.2019.00060)
Supplement: Supplementary file 4 [file Data_Sheet_4.docx]

Supplementary Data 4

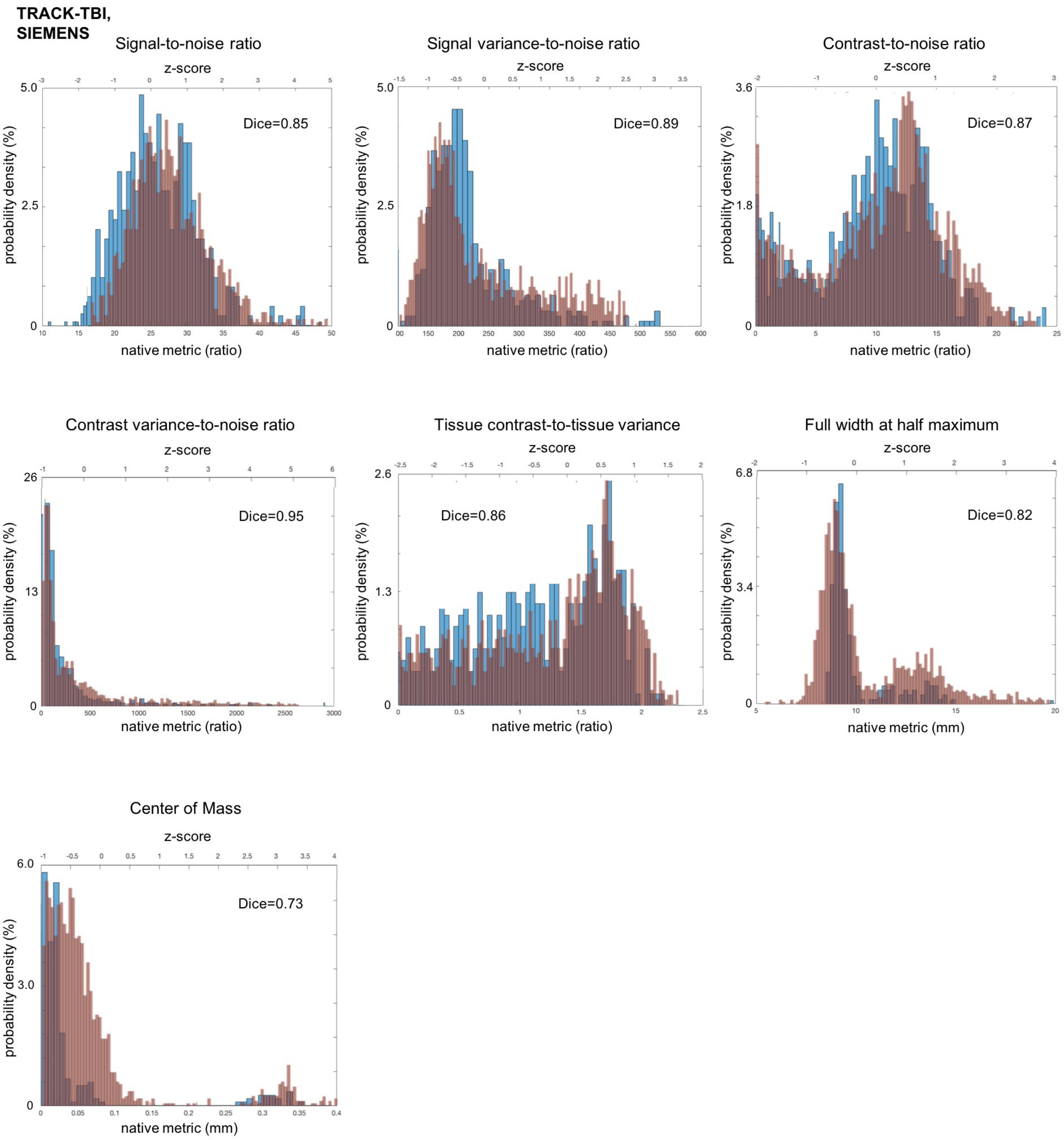

//
